# New insights into the biodiversity of coliphages in the intestine of poultry

**DOI:** 10.1038/s41598-020-72177-2

**Published:** 2020-09-16

**Authors:** Patricia E. Sørensen, Wim Van Den Broeck, Kristoffer Kiil, Dziuginta Jasinskyte, Arshnee Moodley, An Garmyn, Hanne Ingmer, Patrick Butaye

**Affiliations:** 1 Department of Pathology, Bacteriology and Poultry Diseases, Ghent University, Merelbeke, Belgium; 2 Department of Biomedical Sciences, Ross University School of Veterinary Medicine, Basseterre, Saint Kitts and Nevis; 3 Department of Morphology, Ghent University, Merelbeke, Belgium; 4 Department of Bacteria, Parasites and Fungi, Statens Serum Institut, Copenhagen, Denmark; 5 Department of Veterinary and Animal Sciences, University of Copenhagen, Frederiksberg C, Denmark; 6 CGIAR Antimicrobial Resistance Hub, International Livestock Research Institute, Nairobi, Kenya

**Keywords:** Bacteriophages, Biodiversity

## Abstract

Despite phages’ ubiquitous presence and great importance in shaping microbial communities, little is known about the diversity of specific phages in different ecological niches. Here, we isolated, sequenced, and characterized 38 *Escherichia coli*-infecting phages (coliphages) from poultry faeces to gain a better understanding of the coliphage diversity in the poultry intestine. All phages belonged to either the *Siphoviridae* or *Myoviridae* family and their genomes ranged between 44,324 and 173,384 bp, with a G+C content between 35.5 and 46.4%. Phylogenetic analysis was performed based on single “marker” genes; the terminase large subunit, portal protein, and exonucleases, as well as the full draft genomes. Single gene analysis resulted in six distinct clusters. Only minor differences were observed between the different phylogenetic analyses, including branch lengths and additional duplicate or triplicate subclustering. Cluster formation was according to genome size, G+C content and phage subfamily. Phylogenetic analysis based on the full genomes supported these clusters. Moreover, several of our *Siphoviridae* phages might represent a novel unclassified phage genus. This study allowed for identification of several novel coliphages and provides new insights to the coliphage diversity in the intestine of poultry. Great diversity was observed amongst the phages, while they were isolated from an otherwise similar ecosystem.

## Introduction

Bacteriophages (phages) are viruses that have the ability to specifically infect bacteria. They are estimated to be the most abundant form of life on Earth (~ 10^31^ organisms) and can be found in almost every ecosystem, including soil, wastewater, sewage water, seawater and in and on humans and animals^1–4^. Phages are thought to play essential roles in shaping the microbial ecology, including driving the diversity of the bacterial communities^5^. As no single gene is present in all phages, their taxonomic classification is based on host-range, physical characteristics, including size and morphology, genetic structure and composition, and overall genome similarity^6,7^. The phage classification scheme is regularly updated, refined and approved by the International Committee on the Taxonomy of Viruses (ICTV)^8^. Furthermore, in recent years several genome-based phage taxonomy schemes have been proposed^7,9^. According to the National Center for Biotechnology information (NCBI), as of February 2020, 9,238 complete phage genomes have been sequenced. However, despite a continuously rising number of sequenced phage genomes, most of them remain unclassified and poorly characterized. According to the ICTV, a phage genus can be defined as a group of viruses with > 50% nucleotide sequence similarity, which is distinct from viruses of other genera. Moreover, defining characteristics can be determined for each genus, including average genome length and number of coding sequences (CDSs), percentage of shared CDSs, and the presence of specific signature genes in genus members^10^.

Most phages that infect *Escherichia coli*, coliphages, belong the highly heterogeneous *Caudovirales* order, which constitute ~ 96% of all known isolated phages^11^. This order contains five families of tailed phages with dsDNA genomes: *Myoviridae, Siphoviridae, Podoviridae, Ackermannviridae* and *Herelleviridae*^12^. According to ICTV taxonomy (data of February 2020), these families comprise five, eleven, three, two, and five subfamilies, respectively, and 87, 210, 48, three, and 15 genera, respectively. The currently analysed *Myoviridae* coliphages belong to four subfamilies, including *Ounavirinae*, *Peduovirinae*, *Tevenvirinae*, and *Vequintavirinae*, and 17 genera. *Siphoviridae* coliphages are found in only two subfamilies; *Guernseyvirinae* and *Tunavirinae*, and in 13 genera. *Podoviridae* coliphages belong to two subfamilies, the *Autographivirinae* and the *Sepvirinae*, and to ten genera. *Ackermannviridae* coliphages belong only to the *Cvivirinae* subfamily and the *Kuttervirus* genus. To date, there have been no *Herelleviridae* coliphages isolated. To understand the diversity, relationships, and dynamics among any group of phages, nucleotide sequence information is needed^1^. For tailed phages, it has been reported that conserved genes such as the terminase large subunit, the portal protein and major capsid protein, can be used as phylogenetic phylomarkers for the diversity as well as their evolutionary relationship^1,13^.

Compared to their bacterial hosts, relatively few phages have been fully characterized^14^. Besides, despite the phages’ significant role and ubiquitous presence in various areas, little is known on the nature and extent of phage diversity in different ecosystems^3^. Recently, there has been an interest in the diversity of coliphages^1,15,16^. Here, we performed a detailed genome-based characterization and phylogenetic analysis of 38 fully sequenced coliphages, all isolated from a single, relatively unexplored environmental source: poultry faecal material.

## Results

### Phage isolation

In this study, 38 coliphages were isolated from poultry faecal samples collected from 27 Belgian poultry farms located in five different regions, including West Flanders, East Flanders, Antwerp, and Limburg. Between one and seven phages were isolated from each farm using *E. coli* C600 or K514 as host strain.

### Phage morphological analysis

**Figure 1:**
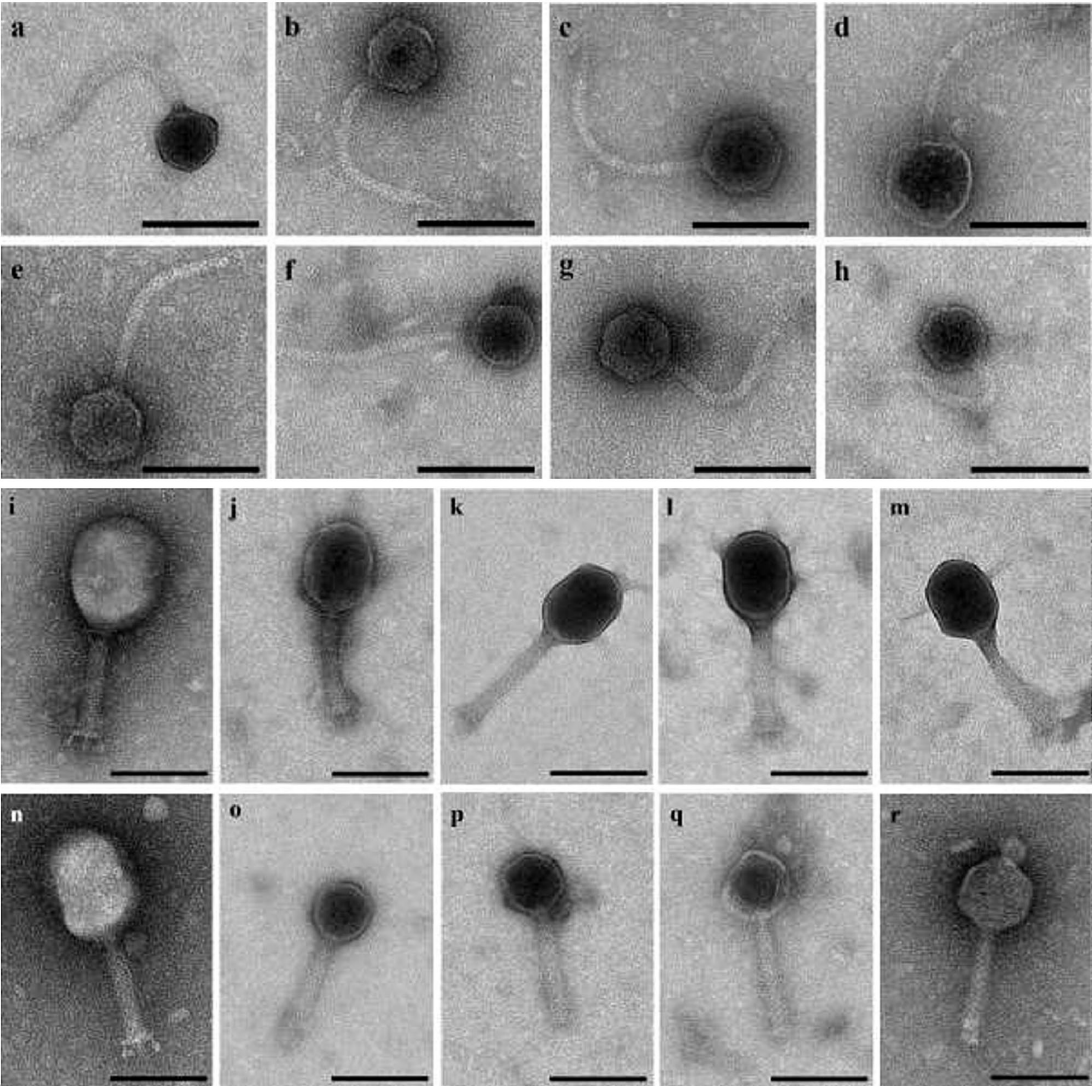
Negative staining electron microscopy images of *Siphoviridae* and *Myoviridae* coliphages. *Siphoviridae* phages: (**a**) Phage 17. (**b**) Phage 53. (**c**) Phage 54. (**d**) Phage 61. (**e**) Phage 70. (**f**) Phage 74. (**g**) Phage 76. (**h**) Phage 77. *Myoviridae* phages: (**i**) Phage 10. (**j**) Phage 11. (**k**) Phage 15. (**l**) Phage 18. (**m**) Phage 30. (**n**) Phage 55. (**o**) Phage 60. (**p**) Phage 62 (**q**) Phage 78. (**r**) Phage 79. The black bars represent 100 nm.

Based on a sequencing cut-off value of ≤ 95% nucleotide similarity, 18 coliphages were selected and subjected to TEM to determine phage morphology and confirm phage classification. Based on the morphological features, the phages were classified into the *Caudovirales* order and either the *Siphoviridae* family or the *Myoviridae* family. Analysis of the isolated *Siphoviridae* phages showed a long flexible non-contractile tail with a length varying between ~ 100 and ~ 200 nm and icosahedral heads with widths ranging from ~ 52 to ~ 77 nm (Fig. 1a–h). Among the isolated *Myoviridae* phages a long straight contractile tail was observed with a tailed length varied between ~ 100 and ~ 120 nm, head widths ranging from ~ 65 to ~ 84 nm, and head lengths from ~ 60 to ~ 110 nm (Fig. 1i–r). Taxonomic classification of each of the coliphages is shown in Table 1.

**Table 1:** Characteristics of the 38 *E. coli* phages investigated in this study.

Phage name	Reg-ion	Farm ID	*E. coli* host	Genome size (bp)	% G+C	# CDSs	Related Ref. phage	Phage family	Phage subfamily	Phage genus	Phage cluster^b^			
											WGS	TLS	PP	Exo
Phage 47	OVL	15	C600	51,063	43.6	83	G29-2	*Sipho*	*Tunavirinae*	*Hanrivervirus*	A_1_	A_1_	A_1_	A_1_
Phage 48	WVL	12	C600	51,031	43.7	85	Henu8	*Sipho*	*Tunavirinae*	*Hanrivervirus*	A_1_	A_1_	A_1_	A_1_
Phage 53	WVL	10	K514	50,835	44.2	87	G29-2	*Sipho*	*Tunavirinae*	*Hanrivervirus*	A_1_	A_1_	A_1_	A_1_
Phage 54	WVL	14	K514	52,602	43.5	88	Henu8	*Sipho*	*Tunavirinae*	*Hanrivervirus*	A_1_	A_1_	A_1_	A_1_
Phage 59	LIM	22	K514	51,702	43.6	85	G29-2	*Sipho*	*Tunavirinae*	*Hanrivervirus*	A_1_	A_1_	A_1_	A_1_
Phage 63	OVL	19	K514	49,132	44.0	79	G29-2	*Sipho*	*Tunavirinae*	*Hanrivervirus*	A_1_	A_2_	A_1_	A_1_
Phage 64	OVL	19	K514	51,352	43.7	85	G29-2	*Sipho*	*Tunavirinae*	*Hanrivervirus*	A_1_	A_1_	A_1_	A_1_
Phage 65	OVL	19	K514	51,031	43.6	83	G29-2	*Sipho*	*Tunavirinae*	*Hanrivervirus*	A_1_	A_1_	A_1_	A_1_
Phage 68	OVL	21	K514	51,291	43.7	84	G29-2	*Sipho*	*Tunavirinae*	*Hanrivervirus*	A_1_	A_1_	A_1_	A_1_
Phage 71	OVL	19	K514	51,446	43.6	85	G29-2	*Sipho*	*Tunavirinae*	*Hanrivervirus*	A_1_	A_1_	A_1_	A_1_
Phage 72	OVL	17	K514	51,284	43.7	84	G29-2	*Sipho*	*Tunavirinae*	*Hanrivervirus*	A_1_	A_1_	A_1_	A_1_
Phage 75	OVL	20	K514	50,445	44.1	88	G29-2	*Sipho*	*Tunavirinae*	*Hanrivervirus*	A_1_	A_1_	A_1_	A_1_
Phage 77	ANT	6	K514	51,073	44.0	85	G29-2	*Sipho*	*Tunavirinae*	*Hanrivervirus*	A_1_	A_1_	A_1_	A_1_
Phage 8	VBR	27	C600	51,031	43.6	83	pSf-1	*Sipho*	*Tunavirinae*	*Hanrivervirus*	A_1_	A_1_	A_1_	A_1_
Phage 28	OVL	19	K514	52,970	44.4	87	SECphi27	*Sipho*	*Tunavirinae*	*Swanvirusa*^a^	A_2_	A_2_	A_2_	A_2_
Phage 56_1	ANT	1	K514	52,716	44.5	86	EcoS-95	*Sipho*	*Tunavirinae*	*Swanvirus*^a^	A_2_	A_2_	A_2_	A_2_
Phage 76	WVL	11	K514	51,905	45.0	93	SECphi27	*Sipho*	*Tunavirinae*	*Swanvirus*^a^	A_2_	A_4_	A_2_	A_2_
Phage 80	OVL	18	K514	52,703	44.5	88	EcoS-95	*Sipho*	*Tunavirinae*	*Swanvirus*^a^	A_2_	A_2_	A_2_	A_2_
Phage 52	WVL	13	K514	53,018	45.9	90	Jahat MG145	*Sipho*	*Tunavirinae*	New genus	A_3_	A_3_	A_3_	A_3_
Phage 56_2	ANT	1	K514	50,829	45.7	87	Jahat MG145	*Sipho*	*Tunavirinae*	New genus	A_3_	A_3_	A_3_	A_3_
Phage 69	WVL	14	K514	62,384	46.3	112	Jahat MG145	*Sipho*	*Tunavirinae*	New genus	A_3_	A_3_	A_3_	A_3_
Phage 17	OVL	19	K514	45,948	44.5	73	CEB_EC3a	*Sipho*	*Tunavirinae*	*Rtpvirus*	B	B	B	B
Phage 58	ANT	4	K514	45,387	44.3	73	CEB_EC3a	*Sipho*	*Tunavirinae*	*Rtpvirus*	B	B	B	B
Phage 70	LIM	25	K514	44,539	44.8	72	CEB_EC3a	*Sipho*	*Tunavirinae*	*Rtpvirus*	B	B	B	B
Phage 73	OVL	17	K514	46,938	44.3	76	CEB_EC3a	*Sipho*	*Tunavirinae*	*Rtpvirus*	B	B	B	B
Phage 74	OVL	21	K514	46,683	44.6	77	CEB_EC3a	*Sipho*	*Tunavirinae*	*Rtpvirus*	B	B	B	B
Phage 61	ANT	3	K514	109,866	39.2	164	T5	*Sipho*	N/A	*Tequintavirus*	C	C	C	C
Phage 60	LIM	26	K514	86,237	39.3	127	Alf5	*Myo*	*Ounavirinae*	*Felixounavirus*	D	D	D	D
Phage 62	VBR	7	K514	87,871	39.0	128	Alf5	*Myo*	*Ounavirinae*	*Felixounavirus*	D	D	D	D
Phage 66	ANT	2	K514	90,196	39.0	137	Alf5	*Myo*	*Ounavirinae*	*Felixounavirus*	D	D	D	D
Phage 78	VBR	8	K514	89,900	39.1	130	Alf5	*Myo*	*Ounavirinae*	*Felixounavirus*	D	D	D	D
Phage 79	LIM	23	K514	89,663	39.0	135	AYO145A	*Myo*	*Ounavirinae*	*Felixounavirus*	D	D	D	D
Phage 15	OVL	21	K514	169,586	37.7	269	MM02	*Myo*	*Tevenvirinae*	*Mosigvirus*	E	E	E	E
Phage 18	OVL	19	K514	169,868	37.7	271	MM02	*Myo*	*Tevenvirinae*	*Mosigvirus*	E	E	E	E
Phage 30	WVL	9	K514	173,384	38.0	275	O157 tp 3	*Myo*	*Tevenvirinae*	*Mosigvirus*	E	E	E	E
Phage 10	ANT	5	K514	168,952	35.5	268	YUEEL01	*Myo*	*Tevenvirinae*	*Tequatrovirus*	F	F	F	F
Phage 11	OVL	16	K514	171,370	35.5	269	fFiEco06	*Myo*	*Tevenvirinae*	*Tequatrovirus*	F	F	F	F
Phage 55	LIM	24	K514	1,699,535	35.6	275	Phage T4	*Myo*	*Tevenvirinae*	*Tequatrovirus*	F	F	F	F

*ANT*, Antwerp; *VBR*, Flemish (Vlaams) Brabant; *WVL*, West Flanders; *OVL*, East Flanders; *LIM*, Limburg; *Sipho*, *Siphoviridae*; *Myo*, *Myoviridae*; *N/A*, No subfamily is defined according to the International Committee on Taxonomy Viruses (ICTV).

^a^*Swanvirus* genus is not yet accepted in the ICTV database^35^.

^b^Phage cluster based on whole genome sequence (WGS), or the single signature genes: terminase large subunit (TLS), portal protein (PP), or exonucleases (Exo).

### Phage genome sequence analysis and annotation

All 38 coliphages isolated in this study were characterized based on WGS data. An overview of the genomic characteristics and properties are listed in Table 1. According to FastQC parameters, good quality of the raw sequence data for all phages was confirmed. The phage genomes ranged in size between 44,324 and 173,384 bp, with a G+C content between 35.5 and 46.4%. Genomes smaller than 90,000 bp had a G+C content between 38.9 and 46.4%, whereas the larger genomes had a G+C content of 35.5–38%. For each coliphage, 72–275 putative CDSs were identified using both automatic and manual annotation. CDSs encoding the phage terminase small subunit, the phage terminase large subunit, the phage portal protein, and phage capsid and scaffold proteins were identified within all 38 coliphage genome sequences. They presented the same conserved genome structure with a general gene order: the terminase small subunit upstream from the terminase large subunit, the phage portal protein and two genes encoding phage capsid and scaffold proteins. In general, one phage terminase small subunit, one phage portal protein, and up to four phage capsid and scaffold proteins were found within each of the phage genomes. Besides, phage exonucleases were identified in all phage genomes. For each phage, one to three CDSs for exonucleases were found. No gene encoding for an integrase was found, indicating that these phages are strictly virulent/lytic phages. No known acquired resistance or virulence genes were detected in any of the 38 phage genomes.

### Phage phylogeny and taxonomy

Taxonomic classification of the 38 isolated coliphages was performed through multiple WGS genome comparisons. These coliphages included 27 (71%) *Siphoviridae* coliphages and 11 (29%) *Myoviridae* coliphages. The *Siphoviridae* phages were compared with 146 published phages from this family. The *Myoviridae* phages were compared with 171 published *Myoviridae* phages. According to ICTV guidelines, phage family, subfamily and genus were predicted based on genome similarity. Results are shown in Table 1. All *Siphoviridae* phages belonged to the *Tunavirinae* subfamily, except for Phage 61. This phage was predicted to belong to the *Tequintavirus* genus, which do not have any ICTV subfamily. The ten phages, Phage 8, 53, 54, 63, 65, 68, 69, 71, 72, and 75 all belonged to the *Hanrivervirus* genus. Three phages belonged to the *Rtpvirus* genus, including Phage 17, 70, and 73. No existing ICTV genus could be assigned to the remaining 13 coliphages. Phage 28, 56_1 and 76 could be assigned to the same unknown genus. Phage 58 and Phage 74 were found to be in the same genus. Phage 47, 48, 59, 64, and 77 were predicted to belong to the same genus. Phage 52, 56_2, and 80 were predicted to belong to the same genus. The 11 *Myoviridae* belonged either to the *Tevenvirinae* or the *Ounavirinae* subfamily. *Tevenvirinae* phages included the six phages: Phage 10, 11, 15, 18, 30 and 55. Phage 10, 11 and 55 belonged to the *Tequatrovirus* genus, and Phage 18 and 30 belonged to the *Mosigvirus* genus. *Ounavirinae* phages included the remaining five phages; Phage 60, 62, 66, 78 and 79. All phages belonged to the *Felixounavirus* genus.

### Phage diversity

**Figure 2:**
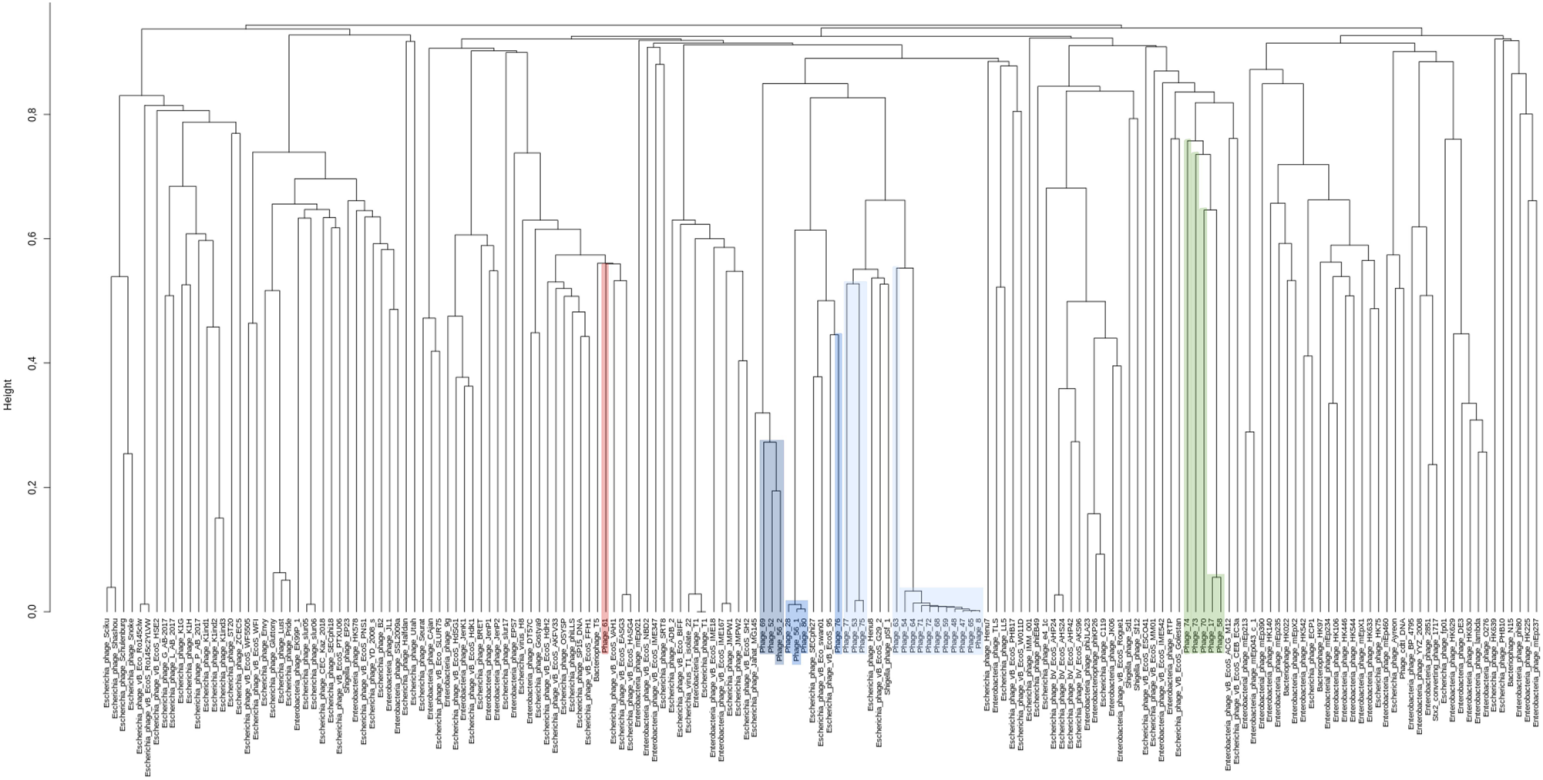
Phylogenetic analysis of *Siphoviridae* coliphages based on WGS sequence. Phages isolated in this study are highlighted. Each colour represents a cluster: Cluster A (blue), cluster B (green), and cluster C (red). Cluster A subclusters include A1 (light blue), A2 (blue), and A3 (dark blue). Distance matrices and clustering are based on kmer length = 10.

**Figure 3:**
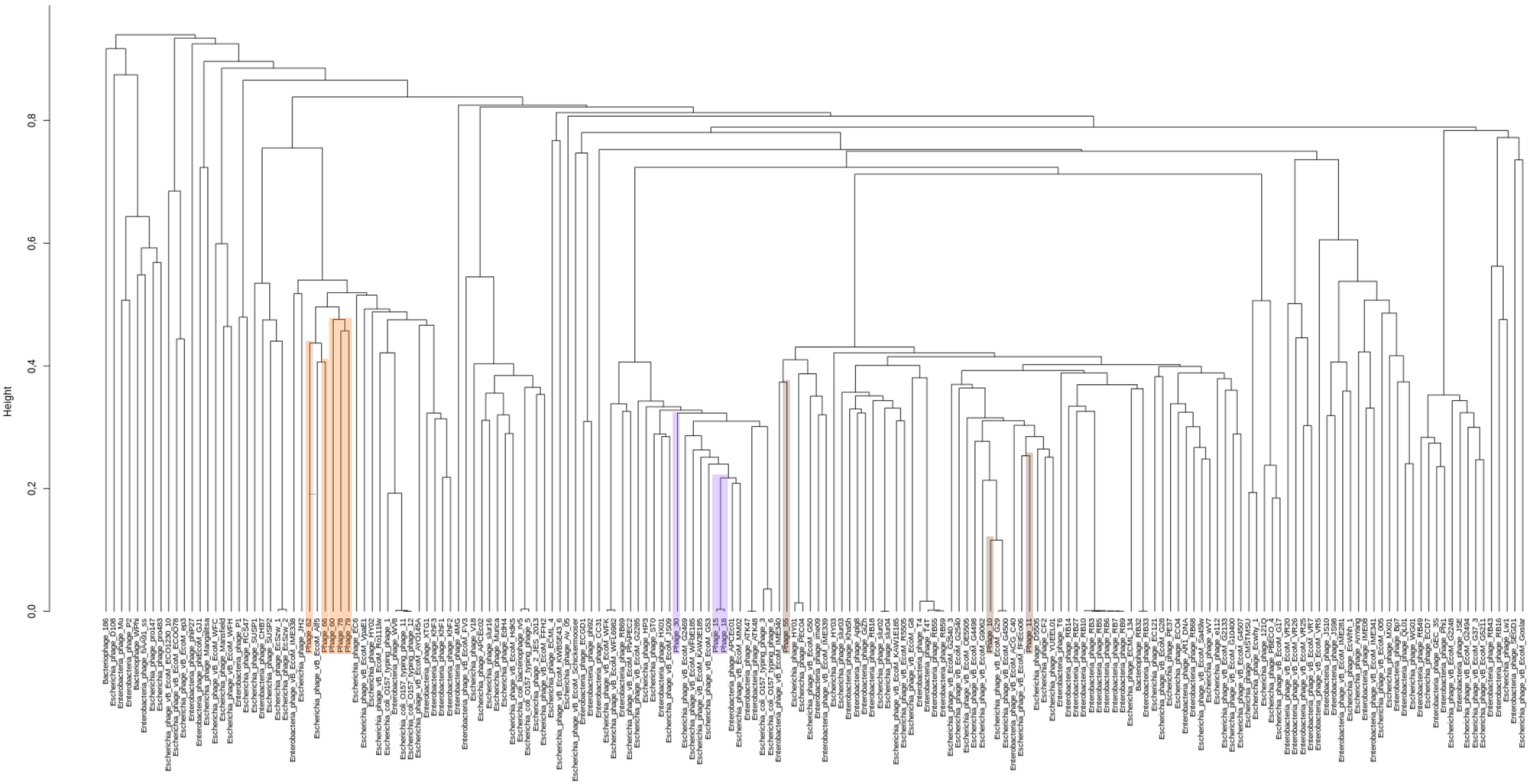
Phylogenetic analysis of *Myoviridae* coliphages based on WGS sequence. Phages isolated in this study are highlighted. Each colour represents a cluster: Cluster D (orange), cluster E (purple), and cluster F (brown). Distance matrices and clustering are based on kmer length = 10.

To investigate the diversity of the coliphages, phage genomes were first clustered based on whole genome sequence. A total of 173 *Siphoviridae* and 182 *Myoviridae* coliphage genomes were included. Characteristics of selected reference genomes are listed in Supplementary Table S1. *Siphoviridae* phages isolated in this study were found in five different (sub)clusters, cluster A1–3, B and C, with a cut-off value of 0.82 (Fig. 2). Cluster A was divided into three subclusters. Fourteen of our phages, formed subcluster A1 together with the three pSf-1-like reference phages from the NCBI database. Phage 80, 28, 56_1, and 76 formed subcluster A2 with the three Swan01-like reference phages. Phages 69, 52, and 56_2 formed subcluster A3 with phage Jahat_MG145. Phage 73, 70, 17, 58 and 74 formed cluster B without any known reference phages. Phage 61 was placed in cluster C with 13 T5-like reference phages. For the *Myoviridae* phages, the resulting phylogeny placed phages isolated in this study in three different clusters with a cut-off height of 0.52 (Fig. 3). Phages 62, 78, 66, 60 and 79 formed cluster D with Felix01-like reference phage Alf5. Phage 30, 15 and 18 formed cluster E with 19 T4-like reference phages (cut-off height of 0.36). Phages 55, 11 and 10 were placed in cluster F with 57 reference phages. At the cut-off height of 0.39 Phage 55 was found in a different subcluster than Phage 10 and 11.

Coliphages were further assessed based on the presence/absence of families of orthologues genes in their pan genome. Similar clusters were observed with only minor changes. For the *Siphoviridae* phage analysis, 5,227 gene groups were included (Supplementary Fig. S1). The resulting phylogenetic analysis placed phages isolated in this study in the same five clusters, cluster A1–3, B and C, with a cut-off height of 0.81 (Supplementary Fig. S2). One additional reference phage was found in cluster B and C, including the T1-like reference phage CEB_EC3a and the T5-like reference phage EPS7, respectively. For the *Myoviridae* phage analysis, 9,420 gene groups were included (Supplementary Fig. S3). The resulting phylogeny placed phages isolated in this study in the same three clusters, cluster D, E, and F, with a cut-off height of 0.58 (Supplementary Fig. S4). For cluster D, additionally 13 Felix01-like reference phages were found. In contrast to the WGS-based analysis, at a cut-off height of 0.39, all cluster F phages isolated in this study, were found in one subcluster with 10 T4-like reference phages. The degree of topological and branch length agreement between the different phylogenetic methods were compared (Supplementary Table S2).

**Figure 4:**
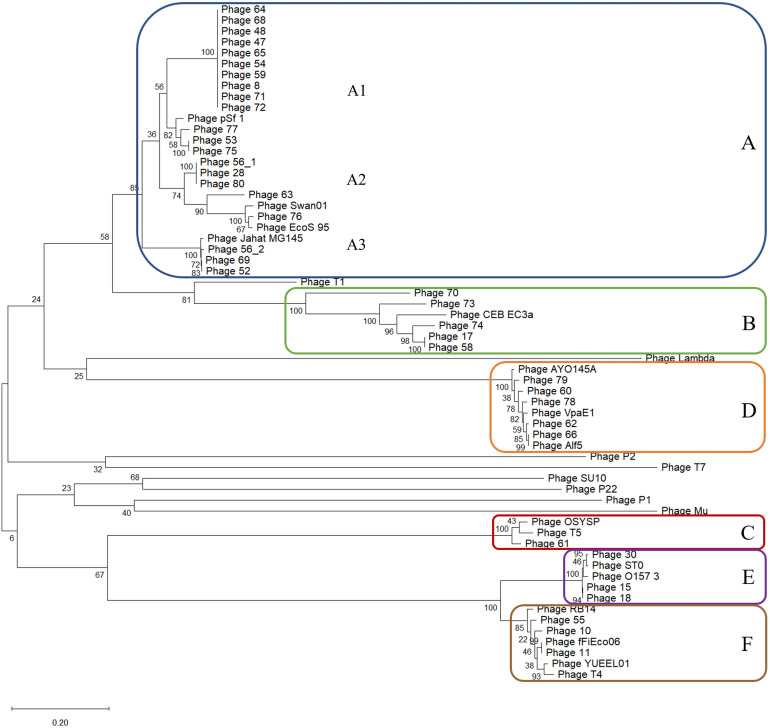
Maximum likelihood tree based on the nucleotide sequences of the phage terminase large subunit. The analysis resulted in six clusters: A–F, according to phage family and subfamily. Cluster A and B: Siphoviridae, Tunavirinae, cluster C: Siphoviridae and Tequintavirus genus, cluster D: Myoviridae, Ounavirinae, and cluster E and F: Myoviridae, Tevenvirinae. Cluster A was divided into three subclusters: A1, A2 and A3. The tree was constructed using the MEGA X software^54^. The percent of data coverage for internal nodes is indicated. The scale bar indicates the number of nucleotide sequence substitutions per site. The analysis included 62 nucleotide sequences, including 24 reference phages listed in Supplementary Table S1 for comparison.

The coliphage diversity was further assessed based on three phage marker genes: the terminase large subunit and phage portal protein, and the phage exonuclease. Selected gene sequences from known phages were included for reference. Results are summarized in Table 1. For all three marker genes, cluster formation was in accordance with resulting clusters of the pan genome- and WGS-based phylogeny, cluster A–F, only with minor differences. Results based on the terminase large subunit analysis are shown below (Fig. 4).

For cluster A, all coliphages isolated in this study were found within same subclusters as for the WGS-based phylogeny except for Phage 63, which was found in the A2 subcluster instead of A1. Analysis based on the phage portal protein resulted in the division of our A2 subcluster phages into two groups: Phage 56_1, 80 and 28 in one group and Phage 76 in the other group (Supplementary Fig. S5). Analysis based on the exonuclease resulted in multiple clusters of cluster C and F, as phages from these clusters encoded 2 or 2–3 exonuclease genes, respectively (Supplementary Fig. S6). Comparison of the cluster construction of the three single genes analysis showed only minor topological and branch length differences (Supplementary Table S2). Moreover, cluster construction was in accordance with phage subfamily defined based on the whole genome. *Siphoviridae* phages from cluster A and B belonged to the *Tunavirinae* subfamily, and *Siphoviridae* phages form cluster C had no defined ICTV subfamily. *Myoviridae* phages from cluster D belonged to the *Ounavirinae* subfamily, and *Myoviridae* phages from cluster E and F belonged to the *Tevenvirinae* subfamily.

### Phage comparative genomics

Pan genome analysis of *Siphoviridae* and *Myoviridae* phages isolated in this study revealed that neither of the two groups had any core genes. Analysis of coliphage genomes from each of the six clusters, A–F, identified core genes (core and softcore) and accessory genes (shell and cloud). As cluster A phages had only five core genes (2% of the total genome), analysis of subclusters, A1, A2 and A3, were performed additionally. Results are summarized in Table 2. The pan genome included between 81 and 333 genes, and core genes constituted between 22 and 73% of the pan genome.

**Table 2:** Overview of comparative genomics analysis.

Cluster	# of phages	Phage names		# of LCBs	# core genes	Core genome	# accessory genes	Accessory genome	Pan genome genes
A1^a^	17	Phage 77, 53, 75, 63, 54, 72, 64, 71, 47, 48, 59, 68, 65, 8, Henu8, G29-2, and pSf-1		16	28	22%	98	78%	126
A2^a^	7	Phage 80, 28, 56_1,76, Swan01, SECphi27 and EcoS_95		7	46	40%	69	60%	115
A3^a^	4	Phages 69, 52, 56_2, and Jahat_MG145		4	62	73%	23	27%	85
B	6	Phage 73, 70, 17, 58, 74, and CEB_EC3a		6	27	33%	54	67	81
C	10	Phage 61, T5, EASG3, HASG4, AKFV33, OSYSP, phiLLS, SP15, FFH1, and HdH2		6–10	86	37%	147	63%	233
D	19	Phage 60, 78, 62, 66, 79, EC6, VpaE1, XTG1, KhF1, KhF2, KhF3, HY02, Ro111lw, O157_1, O157_12, WV8, O157_11, Alf5, and AYO145A		14–17	72	39%	115	61%	187
E	18	Phage 30, 15, 18, HX01, KAW3E185, WFbE185, G53, APCEc01, MM02, HP3, ATK47, ATK48, O157_3, O157_6, ST0, JS09, G2285, and G2469		16–17	184	58%	133	42%	317
F	13	Phage 55, 11, 10, G2540-3, G29, G4500, D5505, G9062, CF2, YUEEL01, fFiEco06, ACG_C40, and OE5505		5	197	59%	136	41%	333

^a^Subcluster instead of cluster. Local collinear blocks (LCB) = indicating homologous DNA regions shared by two or more genomes without sequence rearrangements.

The level of synteny and genomic rearrangement within each cluster or subcluster of related phages was assessed by genome comparison. Results are summarized in Table 2. Eight comparisons were performed, corresponding to the eight (sub)clusters, A1, A2, A3, B, C, D, E, and F resulting from the phage diversity analysis above (Supplementary Fig. S7–S14). Genome comparison of the phages resulted in identification of local collinear blocks (LCBs), indicating homologues DNA regions shared by two or more genomes without sequence rearrangements. The LCBs comprised different modules of genes with different functions, including modules for DNA packaging, structural proteins, head and tail morphogenesis, and host cell lysis. Several modules comprised only hypothetical proteins with unknown function. The average level of conservation varied between the different type of genes.

Genes encoding the terminase large and small subunit, the major capsid protein, DNA primase, single-stranded protein, portal protein, recombinase, specific tail protein and holin were the most conserved genes between all phages, whereas genes with the lowest level of conservation included, specific tail fiber proteins, tail tape measure proteins and HNH homing endonucleases. Hypothetical proteins were found with large variation in level of conservation. Each phage genome comprised between four and 17 LCBs. Genome comparison of phages belonging to subcluster A1, A2 and A3 identified 16, seven and four LCBs, respectively. All phages in each cluster comprised all LCBs. All cluster B phages comprised all six LCBs. For the cluster C phages, between 6 and 10 LCBs were identified for each phage. Phage 61 isolated in this study comprised all nine regions. Variation in number of LCBs was due to a variable repeat region comprising multiple LCBs, which was found only in some of the cluster C phages. For the cluster D comparison, 14–17 different LCBs were identified for each phage. Variation in number of LCBs was due to four different small variable regions of which some of all were missing in the majority of the phages. Phages isolated in this study, including Phage 79, Phage 78, Phage 60, Phage 66, and Phage 62, comprised 17, 17, 16, 15, and 14 LCBs, respectively. Comparison of phages belonging to cluster E identified 18 LCBs. All phages lacked one or both of the same two LCBs. Phages isolated in this study, including Phage 30, Phage 15, and Phage 18, comprised 16, 17, and 17 LCBs, respectively. All 13 cluster F phages included in the comparison comprised all five LCBs. The comparison confirmed the presence of homologue regions between the phages within the clusters but also highlighted that re-arrangement and/or gain/loss of LCBs must have occurred at some point during the evolution of the phages. The region encoding the terminase large subunit and portal protein were present in a conserved region all genomes in all eight comparisons.

## Discussion

In this study, 38 coliphages were isolated from poultry faecal material, sequenced and characterized. The high number of coliphage genomes included in the analysis allowed for a better understanding of both coliphage diversity in poultry and the global coliphage diversity within the *Siphoviridae* and *Myoviridae* families. However, one should be aware of the possible biases as the coliphages were isolated using two *E. coli* K12-derived host strains only and, as such, cannot be seen as the complete coliphage diversity. Both host strains are mutated for the FhuA (previously called TonA) gene, which is used as receptor for some phages, including *Siphoviridae* coliphages T1 and T5^17^. However, phage adsorption is not always restricted to one receptor. Accordingly, we were able to isolate a T5-like phage using the selected host strains. Also, as none of the host strains has the F pili, phages utilising a F pili encoded receptor for absorption, such as *Inoviridae* phages, will most likely not be isolated^17^. The reasons why we could not isolate coliphages from the *Podoviridae* family remains obscure, however, this is in accordance with the finding of Korf et al.^15^, who also did not isolate any *Podoviridae* coliphages from poultry while they could isolate them from sewage and surface water. On the other hand, other studies have successfully isolated *Podoviridae* phages from poultry faeces^18–21^. Several factors might play a role in the type of phage being isolated, including culture and isolation method, host strain, and isolation source. In *Podoviridae* studies mentioned above, phages were isolated using a single or multiple *E. coli* host-strains and the DLA method was similar to this study. However, a notable difference is the type of host-strain used. In our study, laboratory strains were used whereas the *Podoviridae* study host-strains only included *E. coli* strains isolated from poultry. As our 11 *Myoviridae* phages were isolated from samples from 11 different farms, no correlation between phage isolated and geographical location (poultry farm) was found (data not shown). In accordance to the findings of Olsen^16^, the most prevalent genus of the *Myoviridae* and *Siphoviridae* phages isolated in our study was the *Felixounavirus* (45.5%) and the *Hanrivervirus* (33.3%), respectively. Both studies used *E. coli* K-12 derived laboratory strains as host strain for phage isolation. While in a collection of 50 coliphages isolated from surface water, manure, sewage, or animal faeces, 29 different *E. coli* host strains were used^15^. No *Felixounavirus* phages could be isolated and most *Myoviridae* phages belonged to the *Tequatrovirus* genus, followed by the *Mosigvirus* genus. Those two genera were also found in our study.

Currently, the polyphasic approach is the most commonly used for bacterial classification^22,23^, and a similar approach combining multiple methods is recommended when working with phages^12^. However, studying phage taxonomy has proven challenging since no universal conserved marker gene, as the 16S rRNA gene used for bacteria, exists throughout all phage families. Several semi-conserved family-specific marker genes have been proposed as candidates to support taxonomical classification of tailed phages, including DNA packaging and head assembly genes^1,24,25^. Accordingly, in this study we used the terminase large subunit, portal protein and exonucleases as markers, and were able to determine the family and subfamily to all coliphages isolated in this study in accordance with the TEM-based and whole genome-based classification, respectively. Furthermore, the analysis based on the terminase large subunit and portal protein resulted in a clear distinction between the two *Tevenvirinae* clusters, indicating different genera (*Mosigvirus* and *Tequatrovirus*). Thus, single gene-based analysis provides good initial indication in which taxonomic cluster the phages belong to. However, a single gene does not provide a global view of the structural organization of the phage nor accounts for genomic rearrangements, mutations, and mosaicism. Moreover, in accordance with finding from previous studies the selected gene was not always detected in the phage genomes^24^, thus excluding these phages form classification. When using single genes, one should be aware of the possibility of multiple distinct variants of the same gene within one genome. Several of our phages encoded up to three different exonuclease genes. Depending on which gene variant used for analysis, the risk of “false” cluster formation and distance, and hereby a faulty classification at subfamily or genus level was present. Thus, for more comprehensive phage taxonomy, including genus classification, the single gene analysis should be accompanied by whole genome-based analysis as well as functional gene studies. When investigating the evolutionary relationship between phages studies have shown the advantage of combining different proteomic and comparative genomic approaches, including WGS data and well-characterized reference dataset, which take into account the effect of horizontal gene transfer (HGT) and recombination events on the phage genome evolution^7,26^.

In this study, genome-based phylogenetic and taxonomic analysis were performed in combination with traditional morphological examination of the phage using TEM. Through the genome-based analysis we identified a potential new *Siphoviridae* genus. The three unclassified A3 subcluster from this study clustered together with the Jahat_MG145 reference phage, which was a singleton^16^. Thus, we propose that this group of phages represents a new unclassified genus with currently four phages, including Phage 52, Phage 56_2 Phage 69, and Jahat_MG145.

We aimed to expand our knowledge on the coliphage diversity, and observed great diversity among these phages, while they were isolated from a similar ecosystem. The diversity was characterised by a great span in genome size (44.3–173.1 kb) and G+C content range (35.5–46.4%), as well as cluster-specific characteristics of the six phage clusters, A–F. Cluster B phages had the smallest genomes and lowest number of CDSs followed by phages from cluster A, D, C, and E/F. Similar to findings from other studies^16,27^, lower genome size seemed to be correlated with an increase in G+C content. The largest variation in genome size and number of CDSs were observed for the group of *Myoviridae* phages, whereas the largest variation in G+C content (7.2%) was overserved for the group of *Siphoviridae* phages. Notably, the *Tequintavirus* Phage 61 showed to be more similar to the group of *Myoviridae* compared to the other *Siphoviridae* phages based on the above-mentioned characteristics. When omitting Phage 61, G+C content variation for the *Siphoviridae* phages was only 2.9%. Phage G+C content has been shown to be correlated with the G+C content of the phage host^27,28^. Accordingly, differences in G+C content observed for the coliphages might reflect phage-host interactions and co-evolution with past and current host(s). Gene content, including number of core genes appeared to be associated with the cluster. Moreover, number of exonucleases encoded by the phage appeared to be cluster associated as well, as only phages from cluster C and cluster F encoded multiple exonucleases. Encoding multiple exonucleases could be a result of adaptive evolution conferring fitness advantage over other phages. However, it could just as well reflect some of the challenges to accurate phage genome annotation, including false negatives (undetected genes) and incorrect functional annotation^29,30^. Gene content variation has been shown to be related to recombination events resulting in acquisition or loss of gene(s)^31^. Through our comparative genomics analysis of related phages, LCBs with modules with varying level of gene conservation were identified and highlighted the different levels of heterogeneity between different phage clusters. Repeat regions were observed in several of the phage genomes and resulted in variation of LCBs. The presence of these regions should be considered when assessing the gene content variation, as these regions are shown to be prone to genome assembly mistakes, and as such, might represent false level of variation^32^. LCBs modules are also called for mosaic section and the two terms have been used interchangeably throughout history. One definition is that mosaic sections refer to patches of low nucleotide similarity when two similar phage genomes are compared, and that modules refer to exchangeable sections between two or more phages in the population^13^. The genome comparison showed the mosaic nature of the phage genomes, with modules with high level of conservation interspersed with mosaic sections. The mosaic sections could be acquired though HGT from other phages, which is thought to happen when phages are found in the same host. Most often this happens through phage co-infection or single-phage infection of a host that carries one or more prophages^33,34^. Moreover, phages have been shown to acquire genes from their host^35^. The comparative genomics approach hereby underlines the continuous evolution of phage genomes as well as the great phage diversity.

In conclusion, this study has identified a potential new coliphage genus and several new species and provides insight not only to the coliphage diversity of the intestine of poultry but the global coliphage diversity as well. Moreover, classification of phages isolated in this study brings us one step closer to a more refined taxonomic understanding of coliphages. Our comparative genomic analysis showed different levels of heterogeneity between different phage clusters and highlighted the mosaic nature of the phage genomes as well as the continuous evolution of phages in a single environment source. However, to fully understand the complexity and underlying mechanisms of the phage diversity further studies are needed.

## Methods

### Phage isolation and propagation

The phages were isolated from poultry faecal material, collected randomly from 27 poultry houses in Belgium in 2013. Phages were propagated according to Adams and Bonilla et al. with minor modifications^36,37^. Briefly, the samples (5 g) were emulsified in Luria Bertani (LB) broth (Sigma-Aldrich, Saint Louis, MO, USA). The decanted supernatant obtained from each emulsion was enriched by the addition of two early-log phase host bacteria, *E. coli* K-12 derived laboratory strains C600^38^ or K514^39^, a non-restricting, modifying (r_K_−, m_K_+) derivative of strain C600. Suspensions were incubated overnight at 37 °C, with shaking (120 rpm) and were then centrifuged at 4,000 rpm for 30 min to pellet the cellular debris. The supernatant containing the phage(s) was re-centrifuged and filtered using a 0.45 µm membrane filter followed by a 0.2 µm Minisart Filter (Fisher Scientific, Waltham, MA, USA). The enriched phage suspensions were enumerated and tested for lytic activity on the host bacteria using the double-layer agar (DLA) technique^36,40,41^. Briefly, phage suspensions were serial diluted and spotted on an overlay of the respective host bacteria on solid LB medium supplemented with 0.8% agar and 0.5 mM CaCl_2_. A clear zone in the plate, a plaque, resulting from the lysis of host bacterial cells, indicated the presence of virulent coliphage(s). Samples with lytic activity against the indicator strain were further processed for single phage plaque isolation, including three rounds of plaque purification, and propagation. All phage lysates were stored at 4 °C until required.

### Phage morphological analysis

The morphology of unique coliphages (≤ 95% nucleotide similarity) isolated in this study was investigated using transmission electron microscopy (TEM). Phage suspension was applied to the surface of Formvar carbon-coated grids, the phages were fixed using paraformaldehyde (PFA) (4% w/v), washed, and negatively stained with UrAC (1% w/v). After drying, grids were examined using a JEM-1400 Plus transmission electron microscope (JEOL, Benelux).

### Genomic DNA extraction and sequencing

DNA extraction from phage lysates was performed using DNeasy Blood & Tissue Kit (Qiagen, Hilden, Germany) as previously described^42^. The concentration and quality of the DNA was assessed using the NanoDrop (Thermo Scientific, Roskilde, Denmark) and Qubit fluorometer (Thermo Scientific, Roskilde, Denmark) according to the manufacturer’s instructions. Preparation of paired-end 2 × 250 bp sequencing libraries was done using the Nextera XT Kit (Illumina, San Diego, USA) with adaptations for phage genomes as shown elsewhere^43^ and sequenced on the Illumina MiSeq platform using MiSeq Reagent Kit v2 (500-cycles) and manufacturer’s instructions, yielding a total of 16,270–237,128 paired end reads for each phage lysate. Read-pair contigs were generated for each MiSeq cluster prior to assembly.

### Phage genome sequence analysis and annotation

FastQC software (https://www.bioinformatics.babraham.ac.uk/projects/fastqc/), version 0.11.3, was used for quality control validation of the raw reads sequence data. Low-quality sequences were excluded from further analysis. The raw reads were trimmed for quality, adaptor sequences were removed using default parameters. The sequence reads were de novo assembled using QIAGEN Bioinformatics CLC Genomic Workbench, version 11.0.1, using default settings, with minimum contig length changed to 250 bp. An overview of assembly statistics is provided in Supplementary Table S3. The assembled phage sequences were compared with phage homologues from the National Center for Biotechnology Information (NCBI) nucleotide database (https://blast.ncbi.nlm.nih.gov/Blast.cgi) using the Basic Local Alignment Search Tool (BLAST) software^44^, and from the custom PHAge Search Tool Enhanced Release (PHASTER) phage database^45^. Newly assembled phage sequences were compared using both BLAST and PHASTER to identify unique and identical (> 95% nucleotide sequence similarity) phages. Assembled contigs were submitted to the ResFinder database, version 3.2^46^ and the VirulenceFinder database, version 2.0^47^ to identify any acquired antimicrobial resistance and virulence associated genes, respectively. By default, selected threshold for %ID was 90% and 60% for minimum length. All 15 databases for antimicrobial resistance genes were selected. The taxonomic group of *E. coli* bacteria was selected for VirulenceFinder. PHASTER and The Rapid Annotation using Subsystem Technology (RAST) server and the SEED viewer, version 2.0, were used for identification of CDSs and initial annotation of the phage genomes, including identification of the phage terminase large subunit^48^. Gene function of genes defined as “hypothetical protein” was predicted by comparison to homologue genes with defined functions in other related phage genomes. The G+C content of the phages was calculated using the SEED viewer.

### Phage phylogeny and taxonomy

Multiple genome alignment of the WGS sequences was performed using Applied Maths BioNumerics software, version 7.6. According to the ICTV taxonomy guidelines, the 38 coliphages were classified into phage family, subfamily and genera based on nucleotide similarity to known *Siphoviridae* and *Myoviridae* coliphages. Known reference coliphages included were limited to isolates with complete genomes found in the ICTV database and the NCBI database (data of November 2019).

### Phage diversity

Phylogenetic trees based on the phage whole genome sequences were constructed using R, version 3.5.1^49^, for comparison of the coliphages with published *Siphoviridae* or *Myoviridae* reference phage genomes (accessed from the NCBI database). The trees were constructed using unweighted pair group method with arithmetic mean (UPGMA) from a distance matrix of binary distances calculated from either, gene presence/absence within the full genomes of the phages determined using Roary version 3.12.0^50^, Prokka, version 1.13.7^51^ and prodigal, version 2.6.3^52^, or Kmer presence/absence (using 10 and 21mer) based on de novo assembled contigs, as calculated using a python script (Supplementary Script kmer.py).

For phylogenetic analysis based on single marker gene, phage gene sequences were aligned using Clustal X, version 2.1^53^. A maximum likelihood phylogenetic trees (unrooted) was constructed and supported by bootstrap analysis (inferred from 1,000 replicates) with default substitution model (Tamura-Nei model) to assess the diversity of the coliphages using the phylogenetic and molecular evolutionary analyses (MEGA) software, version X^54^. The phylogenetic trees were based on the nucleotide sequences of the CDSs of the following genes: phage terminase large subunit, phage portal protein, or phage exonucleases. The reference genomes included, represented the best matching published sequences to the phages in this study (selected based on the BLAST max score) and core reference genomes for comparison.

The degree of topological and branch length agreement between the different phylogenetic methods and between the three marker genes was investigated using the R packages Analysis of Phylogenetics and Evolution (ape)^55^ and phangorn^56^.

### Phage comparative genomics

A more detailed analysis of the most closely related coliphage genomes was carried out. Genomes were re-annotated using Prokka and pan genome analysis was carried out with Roary using script “roary-e-n-s-p 20-i90*.gff”, including identification of core genome, including core and softcore genes, and accessory genome, including shell and cloud genes.

In order to investigate the level of synteny and genomic rearrangement, whole genome alignment and comparison of coliphages and related reference phages from each cluster or subcluster were performed with the Mauve software using progressiveMauve^57^ with default parameters. No more than 19 of the most related phages were included in each comparison for simplification. Relatedness of the phages were based on percentage of nucleotide similarity and number of shared core genes. Reference genomes were included for annotation references.

## Supplementary information

Supplementary Information.

## Data Availability

Raw reads data for phage genome sequences have been registered with the NCBI BioProject database and assigned BioProject ID: PRJNA631989. Project information are assessible with the following link https://www.ncbi.nlm.nih.gov/bioproject/?term=PRJNA631989. Phage genomes sequences were assigned the following names and NCBI-Sequence Read Achieve (SRA) accession numbers: Phage 8: Escherichia phage vB_EcoS-P8 (SRX8360060), Phage 10: Escherichia phage vB_EcoM-P10 (SRX8360061), Phage 11: Escherichia phage vB_EcoM-P11 (SRX8360071), Phage 15: Escherichia phage vB_EcoM-P15 (SRX8360082), Phage 17: Escherichia phage vB_EcoS-P17 (SRX8360091), Phage 18: Escherichia phage vB_EcoM-P18 (SRX8360092), Phage 28: Escherichia phage vB_EcoS-P28 (SRX8360093), Phage 30: Escherichia phage vB_EcoM-P30 (SRX8360094), Phage 47: Escherichia phage vB_EcoS-P47 (SRX8360095), Phage 48: Escherichia phage vB_EcoS-P48 (SRX8360096), Phage 52: Escherichia phage vB_EcoS-P52 (SRX8360062), Phage 53: Escherichia phage vB_EcoS-P53 (SRX8360063), Phage 54: Escherichia phage vB_EcoS-P54 (SRX8360064), Phage 55: Escherichia phage vB_EcoM-P55 (SRX8360065), Phage 56_1: Escherichia phage vB_EcoS-P56_1 (SRX8360066), Phage 56_2: Escherichia phage vB_EcoS-P56_2 (SRX8360066), Phage 58: Escherichia phage vB_EcoS-P58 (SRX8360067), Phage 59: Escherichia phage vB_EcoS-P59 (SRX8360068), Phage 60: Escherichia phage vB_EcoM-P60 (SRX8360069), Phage 61: Escherichia phage vB_EcoS-P61 (SRX8360070), Phage 62: Escherichia phage vB_EcoM-P62 (SRX8360072), Phage 63: Escherichia phage vB_EcoS-P63 (SRX8360073), Phage 64: Escherichia phage vB_EcoS-P64 (SRX8360074), Phage 65: Escherichia phage vB_EcoS-P65 (SRX8360075), Phage 66: Escherichia phage vB_EcoM-P66 (SRX8360076), Phage 68: Escherichia phage vB_EcoS-P68 (SRX8360077), Phage 69: Escherichia phage vB_EcoS-P69 (SRX8360078), Phage 70: Escherichia phage vB_EcoS-P70 (SRX8360079), Phage 71: Escherichia phage vB_EcoS-P71 (SRX8360080), Phage 72: Escherichia phage vB_EcoS-P72 (SRX8360081), Phage 73: Escherichia phage vB_EcoS-P73(SRX8360083), Phage 74: Escherichia phage vB_EcoS-P74 (SRX8360084), Phage 75: Escherichia phage vB_EcoS-P75 (SRX8360085), Phage 76: Escherichia phage vB_EcoS-P76 (SRX8360086), Phage 77: Escherichia phage vB_EcoS-P77 (SRX8360087), Phage 78: Escherichia phage vB_EcoM-P78 (SRX8360088), Phage 79: Escherichia phage vB_EcoM-P79 (SRX8360089), and Phage 80: Escherichia phage vB_EcoS-P80 (SRX8360090).
