# Supplementary material for: New insights into the biodiversity of coliphages in the intestine of poultry
Source: Sci Rep. 2020 Sep 16;10:15220. doi: 10.1038/s41598-020-72177-2 (PMC7494930; doi:10.1038/s41598-020-72177-2)
Supplement: Supplementary file 1 — Supplementary Information. [file 41598_2020_72177_MOESM1_ESM.docx]

**New Insights into the Biodiversity of Coliphages in the Intestine of Poultry - Supplementary information file**

Author list

Patricia E. Sørensen* ^1,2^, Wim Van Den Broeck ^3^, Kristoffer Kiil ^4^, Dziuginta Jasinskyte ^5^, Arshnee Moodley ^5,6^, An Garmyn ^1^, Hanne Ingmer ^5^, and Patrick Butaye ^1,2^

^1^ Department of Pathology, Bacteriology and Poultry diseases, Ghent University, Belgium

^2^ Department of Biomedical Sciences, Ross University School of Veterinary Medicine, St. Kitts, West Indies

^3^ Department of Morphology, Ghent University, Belgium

^4^ Department of Microbiology and Infection Control, Statens Serum Institut, Denmark

^5^ Department of Veterinary and Animal Sciences, University of Copenhagen, Denmark

^6^ CGIAR Antimicrobial Resistance Hub, International Livestock Research Institute, Nairobi, Kenya

Corresponding author

Patricia E. Sørensen: [patricia.sorensen@uget.be](mailto:patricia.sorensen@uget.be)

**Supplementary Script Kmer.py**

#!/usr/bin/env python3

import Utils

import argparse

import sys

import math

import collections

#import timeit

parser=argparse.ArgumentParser(description="Creates a kmer profile from a fastq or fasta file")

parser.add_argument('seq_files',type=str,nargs='+')

parser.add_argument('-l','--length',type=int,default=10)

parser.add_argument('-e','--euclidian',action="store_true")

args=parser.parse_args()

kmerlength=args.length

def kmer_fq(fil):

kmers=dict()

fqs=Utils.FqStream(fh=fil)

for fq in fqs:

for i in range(len(fq)-kmerlength):

try:

kmers[fq.sseq()[i:i+kmerlength]]+=1.0

except KeyError:

kmers[fq.sseq()[i:i+kmerlength]]=1.0

unfounded=list()

for (k,v) in kmers.items():

try:

if kmers[k[::-1].translate(Utils.tr)] + v <3:

unfounded.append(k)

except KeyError:

unfounded.append(k)

for k in unfounded:

del kmers[k]

if args.euclidian:

normalize(kmers)

return kmers

def kmer_fasta(fil):

kmers=dict()

try:

fasta="".join([line.decode().strip() if line.decode()[0]!=">" else "" for line in fil])

except AttributeError:

fasta="".join([line.strip() if line[0]!=">" else "" for line in fil])

for i in range(len(fasta)-kmerlength):

try:

kmers[fasta[i:i+kmerlength]]+=1.0

except KeyError:

kmers[fasta[i:i+kmerlength]]=1.0

if args.euclidian:

normalize(kmers)

return kmers

def normalize(kmers):

s=0.0

for val in kmers.values():

s+=val*val

s=math.sqrt(s)

for k in kmers.keys():

kmers[k]/=s

return kmers

def dist2(kmer1,kmer2):

dist=0.0

(L1,L2)=(sum(kmer1.values()),sum(kmer2.values()))

for (k,v) in kmer1.items():

try:

dist+=min(v,kmer2[k])

except KeyError:

pass

return dist/(min(L1,L2))

def dist(kmer1,kmer2):

dist=0.0

ignore=set()

for (k,v) in kmer1.items():

try:

d=v-kmer2[k]

dist+=d*d

ignore.add(k)

except KeyError:

dist+=v*v

remainder=set(kmer2.keys()).difference(ignore)

for k in remainder:

d=kmer2[k]

dist+=d*d

return math.sqrt(dist)

kmers=list()

print("Reading data...",file=sys.stderr)

for filename in args.seq_files:

fil=Utils.gzopen(filename)

line=next(fil)

try:

line=line.decode()

except AttributeError:

pass

if line.startswith(">"):

filetype="Fasta"

kmers.append(kmer_fasta(fil))

elif line.startswith("@"):

filetype="Fastq"

fil.seek(0)

kmers.append(kmer_fq(fil))

else:

print("Unrecognized file format:\n{}".format(line),file=sys.stdout)

fil.close()

print("Done",file=sys.stderr)

for i in range(len(kmers)):

print(args.seq_files[i],end="\t")

for j in range(i):

print("\t",end="")

for j in range(i+1,len(kmers)):

print("\t{}".format(dist(kmers[i],kmers[j]) if args.euclidian else dist2(kmers[i],kmers[j])),end="")

print("")

**Supplementary** **Table S1 | Characteristics of reference genomes mentioned in this study**

| **Phage name** | **Phage** | **Genome size (bp)** | **G+C%** | **# CDSs** | **Phage family** | **Phage subfamily** | **Phage cluster** | **Phage genus** | **Accession number** |
| --- | --- | --- | --- | --- | --- | --- | --- | --- | --- |
| pSf-1 | *Shigella* phage pSf-1 | 51821 | 44.0 | 93 | *Siphoviridae* | *Tunavirinae* | A_1_ | *Hanrivervirus* | KC710998.1 |
| YUEEL01 | *Escherichia*  phage YUEEL01 | 169621 | 35.4 | 268 | *Myoviridae* | *Tevenvirinae* | F | *Tequatrovirus* | KY290975.2 |
| fFiEco06 | *Escherichia*  phage vB_EcoM-fFiEco06 | 167076 | 35.5 | 267 | *Myoviridae* | *Tevenvirinae* | F | *Tequatrovirus* | MG781190.1 |
| CEB_EC3a | *Escherichia*  phage vB_Ecos_CEB_EC3a | 44234 | 44.2 | 72 | *Siphoviridae* | *Tunavirinae* | B | *Rtpvirus* | KY398841.1 |
| ST0 | *Escherichia*  phage ST0 | 170496 | 37.7 | 270 | *Myoviridae* | *Tevenvirinae* | E | *Mosigvirus* | MF044457.1 |
| O157_ 3 | *Escherichia*  coli O157 typing phage 3 | 168733 | 37.3 | 268 | *Myoviridae* | *Tevenvirinae* | E | *Mosigvirus* | KP869101.1 |
| Swan01 | *Escherichia*  phage vB_Eco_swan01 | 50865 | 44.7 | 85 | *Siphoviridae* | *Tunavirinae* | A_2_ | Unclassified | LT841304.1 |
| RB14 | Enterobacteria phage RB14 | 165429 | 35.3 | 264 | *Myoviridae* | *Tevenvirinae* | F | *Tequatrovirus* | FJ839692.1 |
| Alf5 | *Escherichia*  phage vB_EcoM_Alf5 | 87662 | 39.0 | 130 | *Myoviridae* | *Ounavirinae* | C | *Felixounavirus* | KX377933.1 |
| OSYSP | *Escherichia*  phage OSYSP | 110901 | 39.2 | 161 | *Siphoviridae* | N/A | D | *Tequintavirus** | MF402939.1 |
| VpaE1 | *Escherichia*  phage vB_EcoM-VpaE1 | 88403 | 38.9 | 126 | *Myoviridae* | *Ounavirinae* | C | *Felixounavirus* | KM657822.1 |
| AYO145A | *Escherichia*  phage vB_EcoM_AYO145A | 87372 | 39.0 | 128 | *Myoviridae* | *Ounavirinae* | C | *Felixounavirus* | KR014248.1 |
| SU10 | *Escherichia*  Phage vB_EcoP_SU10 | 77327 | 42.1 | 127 | *Podoviridae* | N/A | - | *Kuravirus** | KM044272.1 |
| EcoS_95 | *Escherichia*  phage vB_EcoS-95 | 50910 | 44.8 | 87 | *Siphoviridae* | Unclassified | A_2_ | Unclassified | MF564201.1 |
| Jahat_MG145 | *Escherichia*  phage Jahat_MG145 | 50984 | 45.7 | 87 | *Siphoviridae* | *Tunavirinae* | A_3_ | Unclassified | MK552105.1 |
| Lambda | Enterobacteria phage lambda | 48502 | 49.9 | 71 | *Siphoviridae* | *Tunavirinae* | - | *Lambdavirus* | J02459.1 |
| T1 | Enterobacteria phage T1 | 48836 | 45.6 | 76 | *Siphoviridae* | *Tunavirinae* | B | *Tunavirus* | AY216660.1 |
| T4 | Enterobacteria phage T4 | 168903 | 35.3 | 266 | *Myoviridae* | *Tevenvirinae* | F | *Tequatrovirus* | AF158101.6 |
| T5 | Bacteriophage T5 | 121750 | 39.3 | 172 | *Siphoviridae* | N/A | D | *Tequintavirus** | AY543070.1 |
| T7 | Enterobacteria phage T7 | 39937 | 48.4 | 52 | *Podoviridae* | *Autographivirinae* | - | *Teseptimavirus* | NC_001604.1 |
| P1 | *Escherichia*  virus P1 | 66750 | 47.6 | 92 | *Myoviridae* | N/A | - | *Punavirus** | MH445381.1 |
| P2 | Enterobacteria phage P2 | 31200 | 52.6 | 49 | *Myoviridae* | *Peduovirinae* | - | *Peduovirus* | NC_041848.1 |
| P22 | Enterobacteria phage P22 | 41724 | 47.1 | 68 | *Podoviridae* | N/A | - | *Lederbergvirus** | NC_002371.2 |
| Mu | Enterobacteria phage Mu | 36717 | 52.1 | 54 | *Myoviridae* | N/A | - | *Muvirus** | NC_000929.1 |
| Henu8** | *Escherichia*  phage Henu8 | 49890 | 44.2 | 85 | *Siphoviridae* | *Tunavirinae* | A_1_ | *Hanrivervirus* | MN055691.1 |
| **Supplementary** **Table S3 \|** *(Continued)* | | | | | | | | | |
| **Phage name** | **Phage** | **Genome size (bp)** | **G+C%** | **# CDSs** | **Phage family** | **Phage subfamily** | **Phage cluster** | **Phage genus** | **Accession number** |
| G29_2** | *Escherichia*  phage vB_EcoS_G29-2 | 51739 | 44.0 | 89 | *Siphoviridae* | *Tunavirinae* | A_1_ | *Hanrivervirus* | MK373798.1 |
| SEC_phi27** | *Escherichia*  phage SECphi27 | 51811 | 44.7 | 87 | *Siphoviridae* | *Tunavirinae* | A_2_ | Unclassified | LT961732.1 |
| ACG_M12 | Enterobacteria phage vB_EcoS_ACG-M12 | 46054 | 44.0 | 77 | *Siphoviridae* | *Tunavirinae* | B | *Rtpvirus* | NC_019404.1 |
| Golestan | *Escherichia*  phage VB_EcoS-Golestan | 44829 | 50.6 | 78 | *Siphoviridae* | *Guernseyvirinae* | (B) | *Kagunavirus* | MG099933.1 |
| MM01 | *Escherichia*  phage vB_EcoS_MM01 | 43157 | 43.8 | 69 | *Siphoviridae* | *Tunavirinae* | (B) | *Rogunavirus* | MK373793.1 |
| VAH1** | *Escherichia*  phage vB_EcoS_VAH1 | 124537 | 38.6 | 206 | *Siphoviridae* | N/A | C | *Tequintavirus** | MK373792.1 |
| EASG3** | *Escherichia*  phage vB_EcoS_EASG3 | 120715 | 39.0 | 181 | *Siphoviridae* | N/A | C | *Tequintavirus** | MK373799.1 |
| HASG4** | *Escherichia*  phage vB_EcoS_HASG4 | 120603 | 39.0 | 182 | *Siphoviridae* | N/A | C | *Tequintavirus** | MK373797.1 |
| AKFV33 | *Escherichia*  phage bV_EcoS_AKFV33 | 108853 | 38.9 | 160 | *Siphoviridae* | N/A | C | *Tequintavirus** | NC_017969.1 |
| phiLLS** | *Escherichia* _phage_phiLLS | 107263 | 39.0 | 156 | *Siphoviridae* | N/A | C | *Tequintavirus** | KY677846.1 |
| SP15** | *Escherichia* _phage_SP15_DNA | 110964 | 39.1 | 160 | *Siphoviridae* | N/A | C | *Tequintavirus** | AP019559.1 |
| FFH1 | *Escherichia*  phage vB_EcoS_FFH1 | 108483 | 39.2 | 155 | *Siphoviridae* | N/A | C | *Tequintavirus** | NC_024139.1 |
| HdH2** | *Escherichia*  phage vB_EcoS_HdH2 | 120120 | 39.3 | 174 | *Siphoviridae* | N/A | C | *Tequintavirus** | MK373796.1 |
| H8 | *Escherichia*  virus H8 | 104373 | 38.8 | 149 | *Siphoviridae* | N/A | C | *Tequintavirus** | NC_042307.1 |
| DT57C | Enterobacteria_phage_DT57C | 108065 | 39.7 | 149 | *Siphoviridae* | N/A | C | *Tequintavirus** | NC_027356.1 |
| Gostya9** | *Escherichia* _phage_Gostya9 | 101665 | 39.4 | 137 | *Siphoviridae* | N/A | C | *Tequintavirus** | MH203051.1 |
| EC6 | *Escherichia*  phage EC6 | 86231 | 38.9 | 137 | *Myoviridae* | *Ounavirinae* | D | *Felixounavirus* | NC_027369.1 |
| JH2 | *Escherichia*  phage JH2 | 87712 | 38.8 | 132 | *Myoviridae* | *Ounavirinae* | D | *Felixounavirus* | NC_029023.1 |
| XTG1** | Enterobacteria phage XTG1 | 89635 | 38.9 | 130 | *Myoviridae* | *Ounavirinae* | D | *Felixounavirus* | KT184316.1 |
| KhF1** | Enterobacteria phage KhF1 | 88356 | 38.8 | 133 | *Myoviridae* | *Ounavirinae* | D | *Felixounavirus* | KT184313.1 |
| KhF2** | Enterobacteria phage KhF2 | 88309 | 38.8 | 131 | *Myoviridae* | *Ounavirinae* | D | *Felixounavirus* | KT184314.1 |
| KhF3** | Enterobacteria phage KhF3 | 88016 | 38.9 | 130 | *Myoviridae* | *Ounavirinae* | D | *Felixounavirus* | KT184315.1 |
| HY02 | *Escherichia*  phage HY02 | 86252 | 38.9 | 124 | *Myoviridae* | *Ounavirinae* | D | *Felixounavirus* | NC_028872.1 |
| Ro111lw** | *Escherichia*  phage vB_EcoM-Ro111lw | 86950 | 38.8 | 126 | *Myoviridae* | *Ounavirinae* | D | *Felixounavirus* | MH571750.1 |
| O157_ 1** | *Escherichia*  coli O157 typing phage 1 | 88531 | 38.8 | 135 | *Myoviridae* | *Ounavirinae* | D | *Felixounavirus* | NC_041979.1 |
| O157_ 11** | *Escherichia*  coli O157 typing phage 11 | 88771 | 38.9 | 134 | *Myoviridae* | *Ounavirinae* | D | *Felixounavirus* | KP869109.1 |
| **Supplementary** **Table S1 \|** *(Continued)* | | | | | | | | | |
| **Phage name** | **Phage** | **Genome size (bp)** | **G+C%** | **# CDSs** | **Phage family** | **Phage subfamily** | **Phage cluster** | **Phage genus** | **Accession number** |
| O157_ 12** | *Escherichia*  coli O157 typing phage 12 | 88632 | 38.9 | 132 | *Myoviridae* | *Ounavirinae* | D | *Felixounavirus* | KP869110.1 |
| WV8 | Enterobacteria phage WV8 | 88487 | 38.9 | 134 | *Myoviridae* | *Ounavirinae* | D | *Felixounavirus* | NC_012749.1 |
| HX01 | Enterobacteria phage HX01 | 169158 | 37.6 | 268 | *Myoviridae* | *Tevenvirinae* | E | *Mosigvirus* | NC_018855.1 |
| KAW3E185** | *Escherichia*  phage vB_EcoM_KAW3E185 | 170187 | 37.6 | 270 | *Myoviridae* | *Tevenvirinae* | E | *Mosigvirus* | MK373782.1 |
| WFbE185** | *Escherichia*  phage vB_EcoM_WFbE185 | 170429 | 37.6 | 272 | *Myoviridae* | *Tevenvirinae* | E | *Mosigvirus* | MK373778.1 |
| G53** | *Escherichia*  phage vB_EcoM_G53 | 167834 | 37.8 | 268 | *Myoviridae* | *Tevenvirinae* | E | *Mosigvirus* | MK327943.1 |
| APCEc01 | *Escherichia*  phage APCEc01 | 168771 | 37.7 | 267 | *Myoviridae* | *Tevenvirinae* | E | *Mosigvirus* | NC_029091.1 |
| MM02** | *Escherichia*  phage vB_EcoM_MM02 | 169201 | 37.6 | 269 | *Myoviridae* | *Tevenvirinae* | E | *Mosigvirus* | MK373784.1 |
| HP3 | *Escherichia*  phage HP3 | 170254 | 37.6 | 268 | *Myoviridae* | *Tevenvirinae* | E | *Mosigvirus* | NC_041920.1 |
| ATK47** | Enterobacteria phage ATK47 | 170020 | 37.6 | 270 | *Myoviridae* | *Tevenvirinae* | E | *Mosigvirus* | KT184309.1 |
| ATK48** | Enterobacteria phage ATK48 | 169729 | 37.6 | 270 | *Myoviridae* | *Tevenvirinae* | E | *Mosigvirus* | KT184310.1 |
| O157_ 6 | *Escherichia*  coli O157 typing phage 6 | 160570 | 37.6 | 249 | *Myoviridae* | *Tevenvirinae* | E | *Mosigvirus* | NC_041864.1 |
| JS09 | *Escherichia*  phage vB_EcoM_JS09 | 169148 | 37.6 | 271 | *Myoviridae* | *Tevenvirinae* | E | *Mosigvirus* | NC_024124.2 |
| G2285** | *Escherichia*  phage vB_EcoM_G2285 | 166675 | 37.5 | 262 | *Myoviridae* | *Tevenvirinae* | E | *Mosigvirus* | MK327933.1 |
| G2469** | *Escherichia*  phage vB_EcoM_G2469 | 170452 | 37.6 | 271 | *Myoviridae* | *Tevenvirinae* | E | *Mosigvirus* | MK327934.1 |
| G2540_3** | *Escherichia*  phage vB_EcoM_G2540-3 | 168654 | 35.3 | 271 | *Myoviridae* | *Tevenvirinae* | F | *Tequatrovirus* | MK327944.1 |
| G29** | *Escherichia*  phage vB_EcoM_G29 | 168241 | 35.3 | 269 | *Myoviridae* | *Tevenvirinae* | F | *Tequatrovirus* | MK327940.1 |
| G4500** | *Escherichia*  phage vB_EcoM_G4500 | 168363 | 35.3 | 268 | *Myoviridae* | *Tevenvirinae* | F | *Tequatrovirus* | MK327945.1 |
| D5505** | *Escherichia*  phage D5505 | 168049 | 35.4 | 269 | *Myoviridae* | *Tevenvirinae* | F | *Tequatrovirus* | MK327929.1 |
| G9062** | *Escherichia*  phage vB_EcoM_G9062 | 168670 | 35.3 | 268 | *Myoviridae* | *Tevenvirinae* | F | *Tequatrovirus* | MK373779.1 |
| CF2 | *Escherichia*  phage CF2 | 168188 | 35.4 | 264 | *Myoviridae* | *Tevenvirinae* | F | *Tequatrovirus* | NC_041919.1 |
| ACG_C40** | Enterobacteria phage vB_EcoM_ACG-C40 | 167396 | 35.2 | 273 | *Myoviridae* | *Tevenvirinae* | F | *Tequatrovirus* | NC_019399.1 |
| OE5505** | *Escherichia*  phage vB_EcoM_OE5505 | 168756 | 35.2 | 273 | *Myoviridae* | *Tevenvirinae* | F | *Tequatrovirus* | MK373785.1 |

*No subfamily is defined according to the International Committee on Taxonomy Viruses (ICTV). Phage genus is used instead. N/A = none applicable. - = singleton, no cluster.

**Not found in ICTV database. Classification according to National Center for Biotechnology information (NCBI).

**Supplementary Table S2 |** **Overview of topological and branch length agreement**

|  |  | *ape package* | | *phangorn package* | | |
| --- | --- | --- | --- | --- | --- | --- |
| **Analysis** | **Tree comparison** | **Topological distance score** | **Robinson-Foulds distance** | **Symmetric difference** | **Branch score difference** | **Path difference** |
| *Siphoviridae* | kmer10 vs. kmer21 | 12.16553 | 324 | 323 | - | 2422.025 |
|  | kmer10 vs. roary | 15.71623 | 329 | 241 | - | 2683.053 |
|  | kmer21 vs. roary | 15.26434 | 333 | 348 | - | 2713.845 |
| *Myoviridae* | kmer10 vs. kmer21 | 16.67333 | 346 | 346 | - | 2224.267 |
|  | kmer10 vs. roary | 12.56981 | 340 | 359 | - | 3640.188 |
|  | kmer21 vs. roary | 15.81139 | 346 | 365 | - | 3666.915 |
| Single genes | TLS vs. PP | 3.38274 | 102 | 102 | 7.671297 | 272.736136 |
|  | TLS vs. Exo | 3.56119 | 112 | 124 | 7.215488 | 368.323228 |
|  | PP vs. Exo | 2.94990 | 120 | 132 | 6.026867 | 419.485399 |

kmer10 = tree based on kmer (10) presence/absence based on *de novo* assembled contigs. Kmer21 = tree based on kmer (21) presence/absence based on *de novo* assembled contigs. Roary = tree based on gene presence/absence within the full genomes. TLS = terminase large subunit. PP = portal protein. Exo = exonuclease.

The “Topological distance score” is calculated using the dist.topo function (score method).

The “Robinson-Foulds distance” is calculated using the RF.dist function.

The “symmetric difference” is calculated using the treedist function. It is similar to the Robinson-Foulds distance and the Penny and Hendy’s distance.

The “Path difference” is calculated using the treedist function and is the difference in path lengths, counted as the numbers of branches, between the pairs of tips

**Supplementary Table S3 | Overview of assembly settings and statistics**

|  | *Raw data* | | *Trimming* | | *Assembly* | | | | | |
| --- | --- | --- | --- | --- | --- | --- | --- | --- | --- | --- |
| **Phage** | **# of reads** | **Avg. length** | **# of reads after trim** | **Avg. length after trim** | **# of matched reds** | **Min. contig length** | **Max. contig length** | **# Contigs** | **N50** |  |
| Phage 8 | 20854 | 212.9 | 20792 | 199.1 | 20792 | 51031 | 51031 | 1 | 51031 |  |
| Phage 10 | 175980 | 212.0 | 175598 | 212.2 | 174572 | 380 | 167166 | 4 | 167166 |  |
| Phage 11 | 237128 | 197.3 | 236493 | 197.6 | 235800 | 503 | 169478 | 3 | 169478 |  |
| Phage 15 | 78816 | 184.6 | 78377 | 185.2 | 77992 | 169392 | 169392 | 1 | 169392 |  |
| Phage 17 | 28544 | 204.8 | 28.473 | 204.9 | 28300 | 250 | 44592 | 5 | 44592 |  |
| Phage 18 | 122820 | 179.6 | 122040 | 180.3 | 121527 | 477 | 169391 | 2 | 169391 |  |
| Phage 28 | 21654 | 230.5 | 21628 | 230.4 | 21376 | 254 | 52716 | 2 | 52716 |  |
| Phage 30 | 162094 | 187.9 | 161482 | 188.3 | 160623 | 253 | 169109 | 10 | 169109 |  |
| Phage 47 | 32582 | 198.2 | 32462 | 198.4 | 32305 | 958 | 50105 | 2 | 50105 |  |
| Phage 48 | 18862 | 211.8 | 18844 | 210.8 | 18665 | 51031 | 51031 | 1 | 51031 |  |
| Phage 52 | 44154 | 208.7 | 44024 | 208.7 | 43542 | 373 | 50784 | 5 | 50784 |  |
| Phage 53 | 37154 | 210.5 | 37072 | 210.6 | 36883 | 253 | 50582 | 2 | 50582 |  |
| Phage 54 | 62702 | 210.2 | 62541 | 210.3 | 62213 | 251 | 51031 | 5 | 51031 |  |
| Phage 55 | 233558 | 198.8 | 232979 | 199.0 | 231603 | 254 | 167131 | 8 | 167131 |  |
| Phage 56 | 55786 | 209.9 | 55672 | 209.9 | 55010 | 50829 | 52716 | 2 | 52716 |  |
| Phage 58 | 51386 | 209.7 | 51321 | 209.6 | 51069 | 256 | 44592 | 3 | 44592 |  |
| Phage 59 | 16270 | 235.4 | 16258 | 235.3 | 16053 | 671 | 51031 | 2 | 51031 |  |
| Phage 60 | 34730 | 202.1 | 34624 | 202.5 | 34435 | 86237 | 86237 | 1 | 86237 |  |
| Phage 61 | 219040 | 202.5 | 218539 | 202.5 | 217486 | 254 | 108138 | 6 | 108138 |  |
| Phage 62 | 27242 | 201.9 | 27136 | 202.4 | 26952 | 87871 | 87871 | 1 | 87871 |  |
| **Supplementary Table S3 \|** *Continued* | | | | | | | | | |  |
|  | *Raw data* | | *Trimming* | | *Assembly* | | | | |  |
| **Phage** | **# of reads** | **Avg. length** | **# of reads after trim** | **Avg. length after trim** | **# of matched reds** | **Min. contig length** | **Max. contig length** | **# Contigs** | **N50** |  |
| Phage 63 | 50718 | 206.5 | 50616 | 206.5 | 50416 | 49132 | 49132 | 1 | 49132 |  |
| Phage 64 | 44872 | 209.0 | 44758 | 209.1 | 44527 | 321 | 51031 | 2 | 51031 |  |
| Phage 65 | 41890 | 210.1 | 41804 | 210.2 | 41612 | 51031 | 51031 | 1 | 51031 |  |
| Phage 66 | 46548 | 208.5 | 46400 | 209.0 | 46135 | 257 | 89604 | 3 | 89604 |  |
| Phage 68 | 65582 | 211.2 | 65499 | 211.0 | 65188 | 262 | 51029 | 2 | 51029 |  |
| Phage 69 | 48632 | 214.9 | 48532 | 214.8 | 47924 | 278 | 50777 | 22 | 50777 |  |
| Phage 70 | 45032 | 212.0 | 44934 | 212.1 | 44711 | 250 | 44290 | 2 | 44290 |  |
| Phage 71 | 24156 | 230.6 | 24128 | 230.7 | 23787 | 415 | 51031 | 2 | 51031 |  |
| Phage 72 | 32508 | 222.4 | 32442 | 222.5 | 32100 | 253 | 51031 | 2 | 51031 |  |
| Phage 73 | 42832 | 226.7 | 42752 | 226.8 | 42407 | 46738 | 46738 | 1 | 46738 |  |
| Phage 74 | 61362 | 211.3 | 61208 | 211.5 | 60737 | 258 | 45171 | 4 | 45171 |  |
| Phage 75 | 44444 | 208.3 | 44354 | 208.3 | 44086 | 50445 | 50445 | 1 | 50445 |  |
| Phage 76 | 43978 | 219.5 | 43926 | 219.3 | 43522 | 504 | 50843 | 3 | 50843 |  |
| Phage 77 | 59306 | 215.2 | 59183 | 215.3 | 58920 | 51073 | 51073 | 1 | 51073 |  |
| Phage 78 | 49218 | 206.4 | 49098 | 206.6 | 48677 | 256 | 89644 | 2 | 89644 |  |
| Phage 79 | 50822 | 246.4 | 50816 | 246.4 | 50360 | 253 | 87100 | 7 | 87100 |  |
| Phage 80 | 46730 | 207.1 | 46608 | 207.2 | 46307 | 52703 | 52703 | 1 | 52703 |  |

Default settings was used for CLC Genomics Workbech *de novo* assembly, except for minimum contig length that was changed to 250 bp.


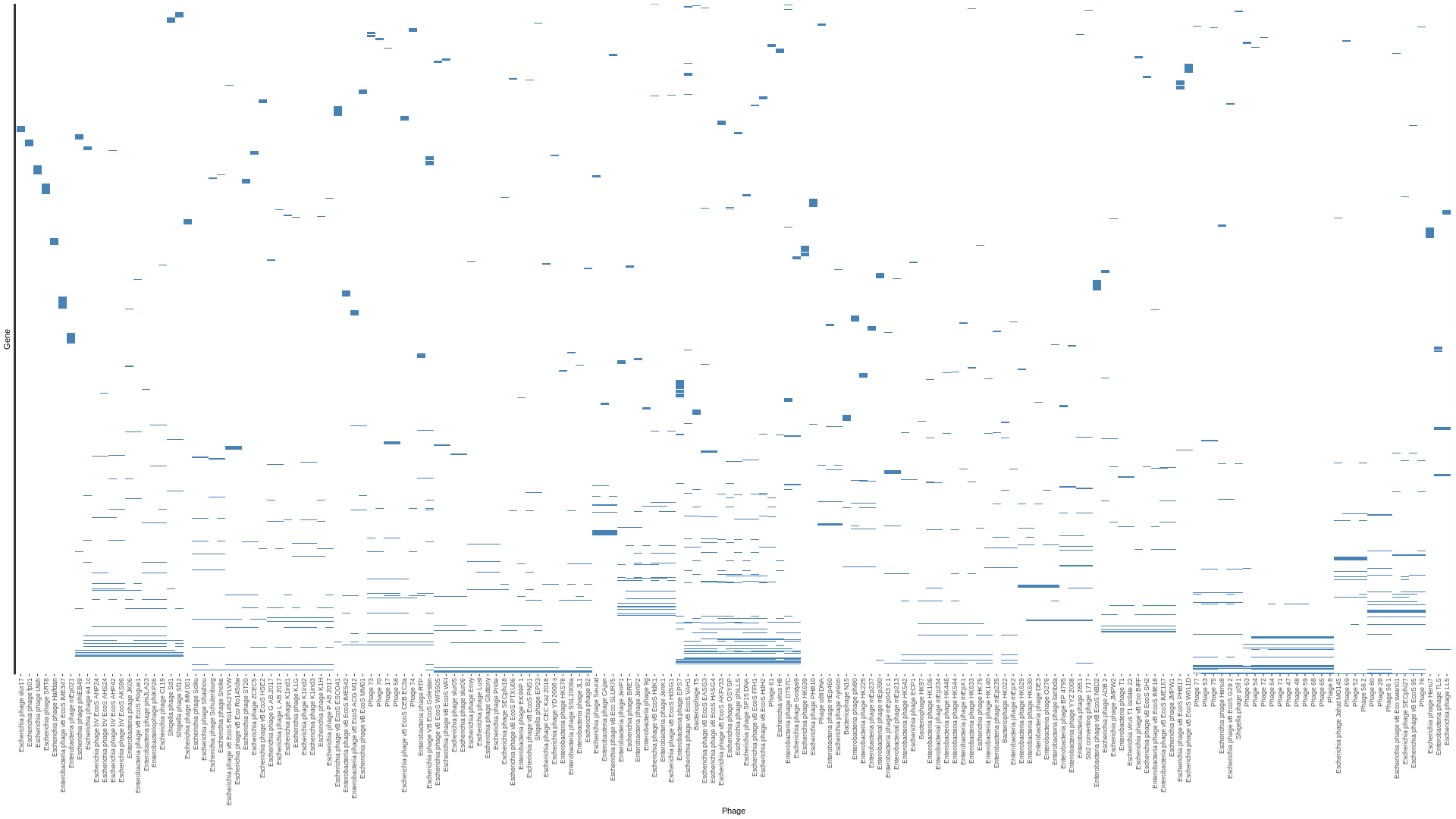


**Supplementary Figure S1 | Roary matrix based on gene list of *Siphoviridae* coliphages.** Phages isolated in this study are highlighted. Each colour represents a cluster: Cluster A (blue), cluster B (green), and cluster C (red). Cluster A subclusters include A1 (light blue), A2 (blue), and A3 (dark blue).


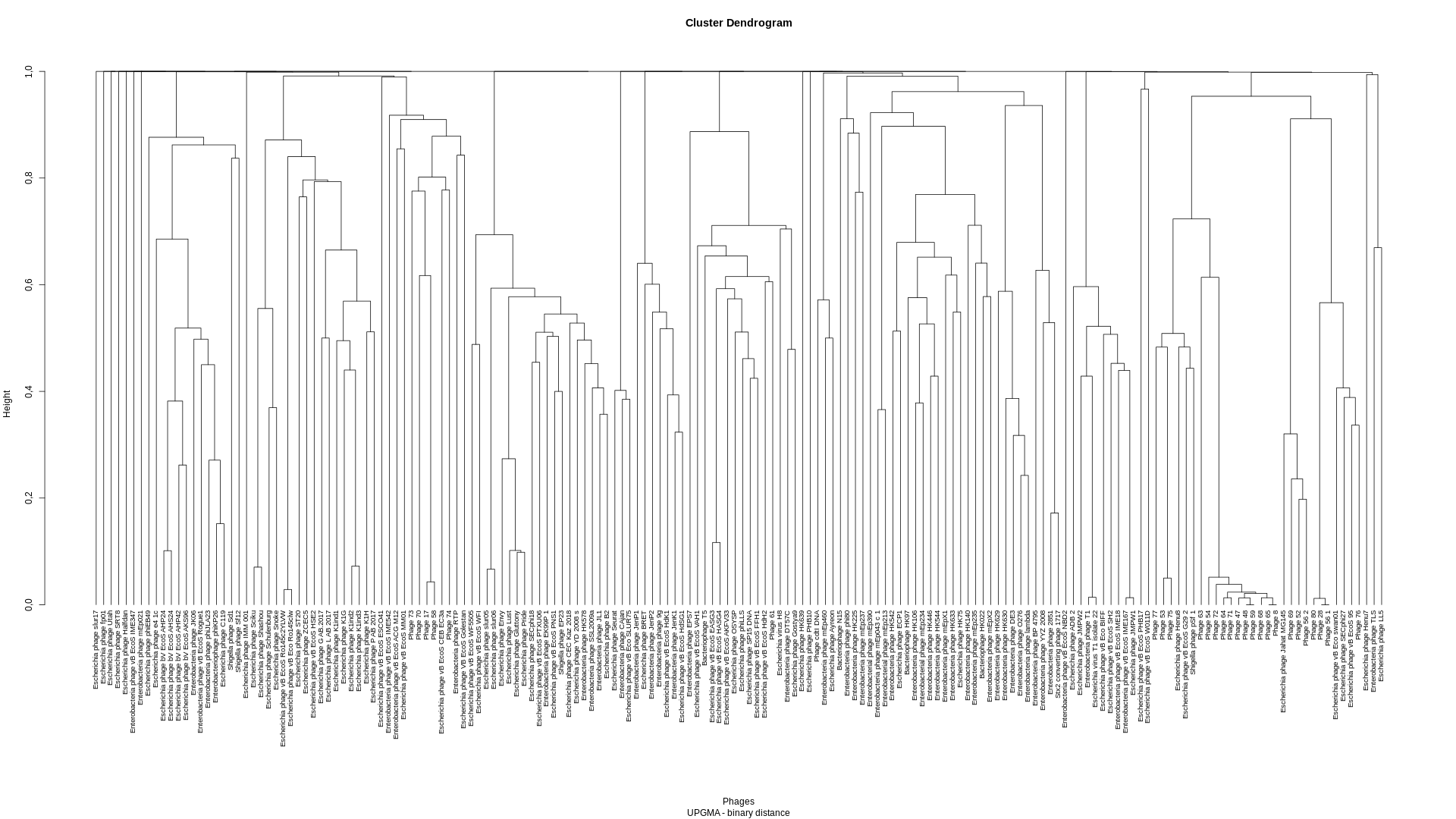


**Supplementary Figure S2 | UPGMA tree based on roary matrix of *Siphoviridae* coliphages**. Phages isolated in this study are highlighted. Each colour represents a cluster: Cluster A (blue), cluster B (green), and cluster C (red). Cluster A subclusters include A1 (light blue), A2 (blue), and A3 (dark blue)

**
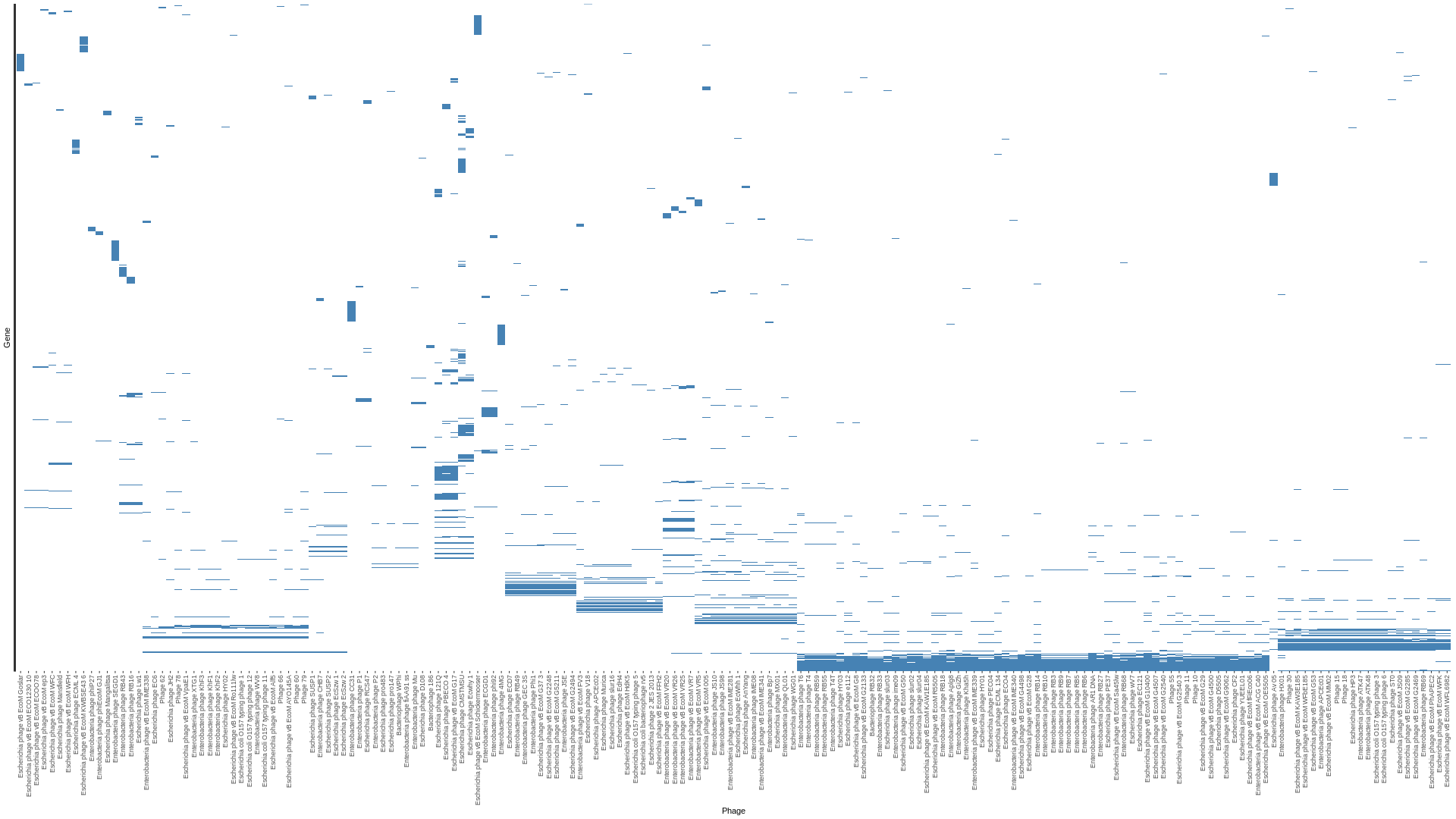
**

**Supplementary Figure S3 | Roary matrix based on gene list of *Myoviridae* coliphages.** Phages isolated in this study are highlighted. Each colour represents a cluster: Cluster D (orange), cluster E (purple), and cluster F (brown).

**
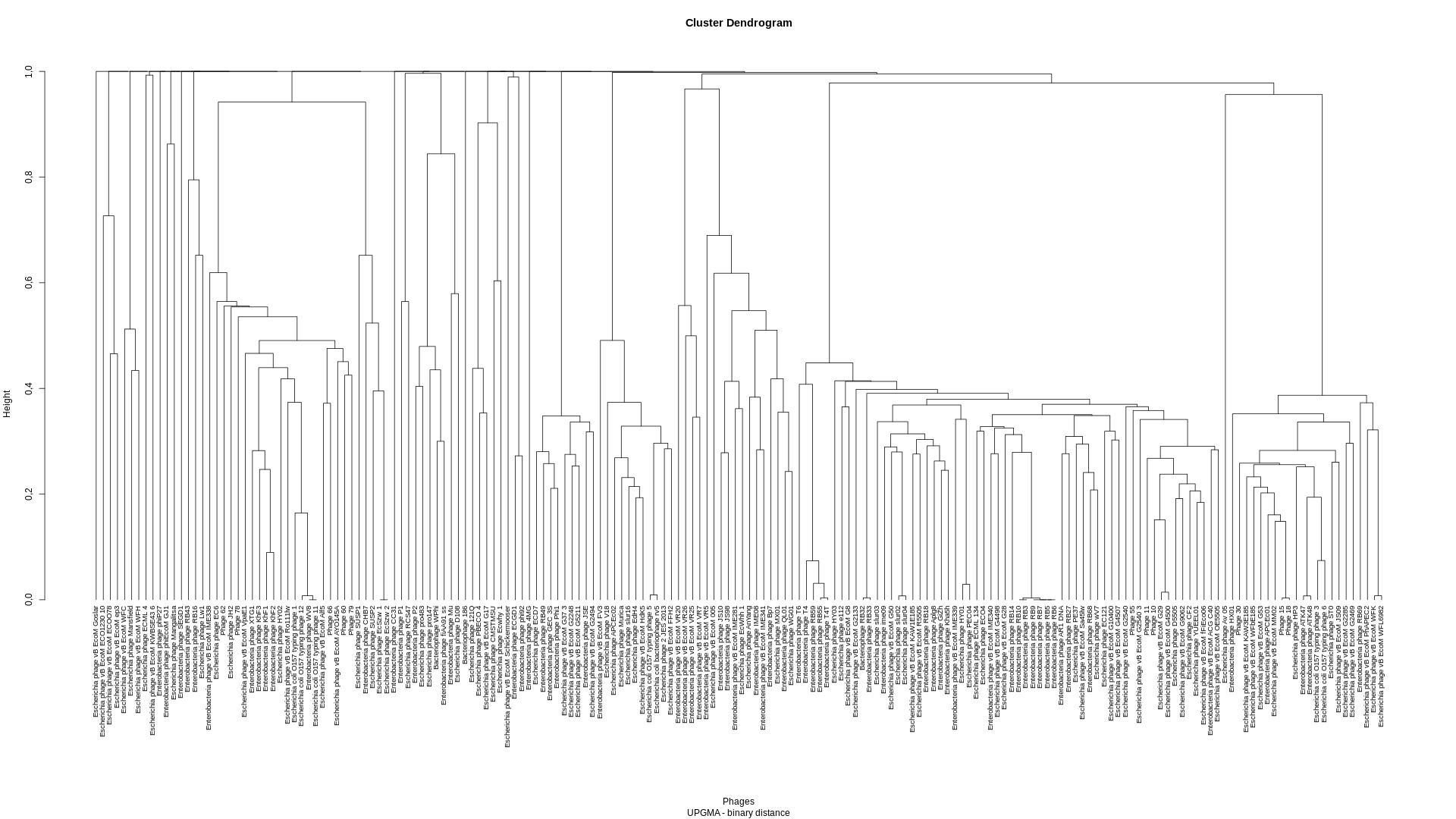

Supplementary Figure S4 | UPGMA tree based on roary matrix of *Myoviridae* coliphages**. Phages isolated in this study are highlighted. Each colour represents a cluster: Cluster D (orange), cluster E (purple), and cluster F (brown).


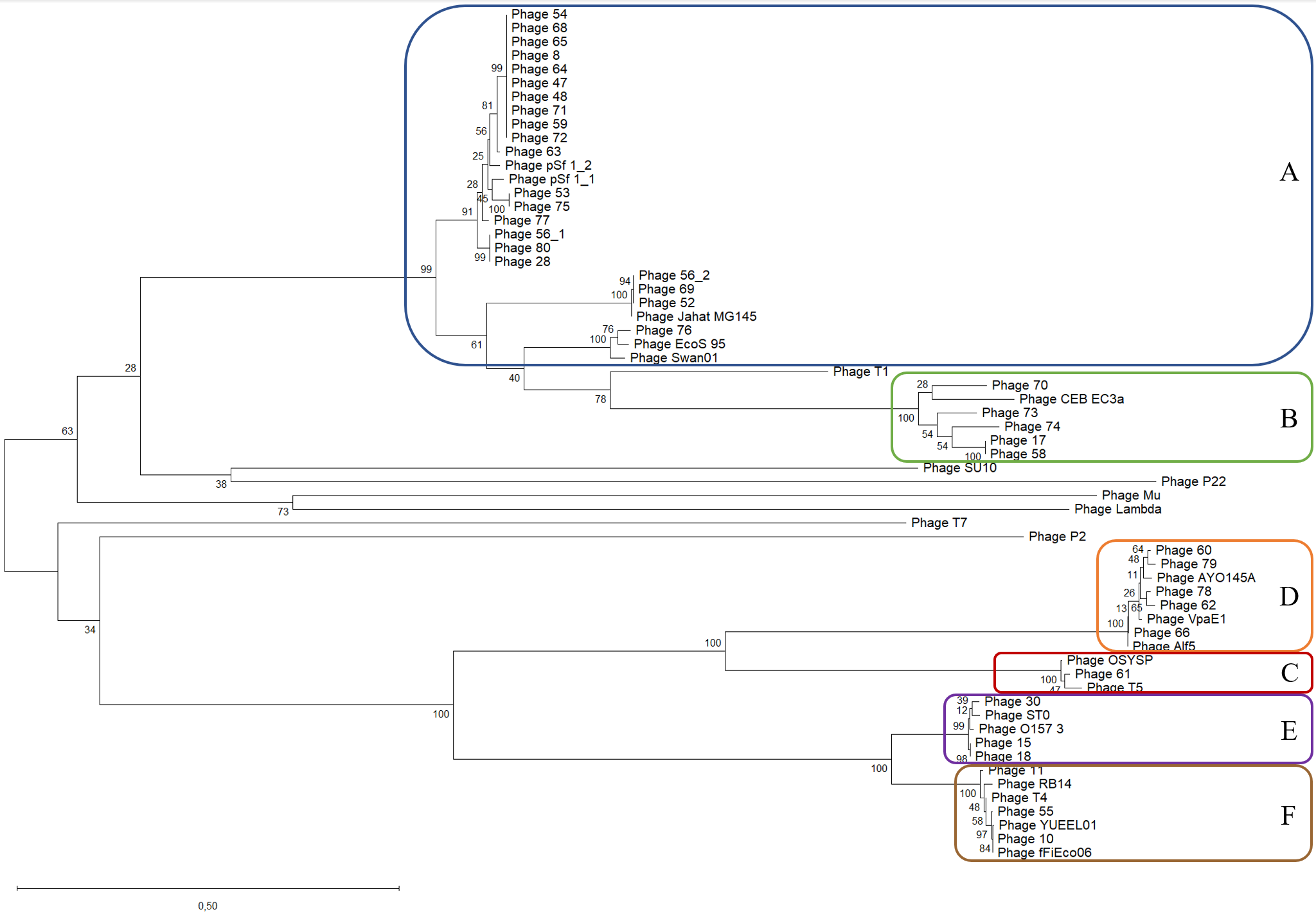


A2

A1

A3

A2

**Supplementary Figure S5 |** **Maximum likelihood tree based on the nucleotide sequence of the phage portal protein.** Phages were grouped together into six clusters: A-F, according to phage family and subfamily. Cluster A and B: *Siphoviridae,* *Tunavirinae*, cluster C: *Siphoviridae* and *Tequintavirus* genus, cluster D: *Myoviridae, Ounavirinae*, and cluster E and F: *Myoviridae*, *Tevenvirinae*. Cluster A was divided into three subclusters: A1, A2 and A3. Subcluster A2 was divided in two. The tree was constructed using the MEGA X software. The percentage of threes in which the associated taxa clustered together is shown next to the branches. The tree is drawn to scale, with branch lengths measured in the number of substitutions per site. The analysis involved 62 nucleotide sequences.


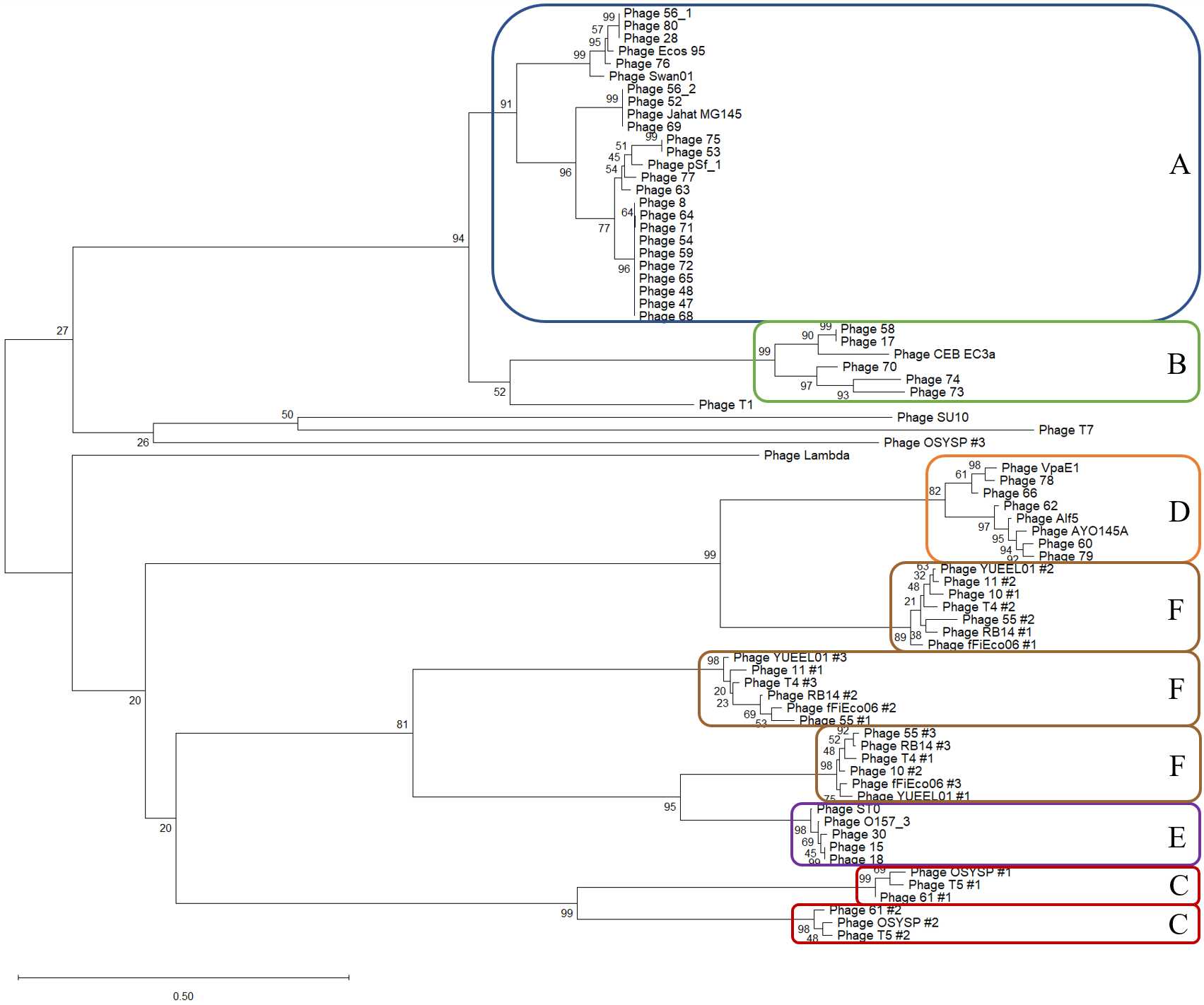


A2

A3

A1

**Supplementary Figure S6 | Maximum likelihood tree based on the nucleotide sequences of phage exonucleases.** The tree comprised six clusters: A-F, according to phage family and subfamily. Cluster A and B: *Siphoviridae,* *Tunavirinae*, cluster C: *Siphoviridae* and *Tequintavirus* genus, cluster D: *Myoviridae, Ounavirinae*, and cluster E and F: *Myoviridae*, *Tevenvirinae*. Cluster A was divided into three subclusters: A1, A2 and A3. Cluster C and F were found in two and three copies, respectively. The tree was constructed using the MEGA X software. The percent of data coverage for internal nodes is indicated. The tree is drawn to scale, with branch lengths measured in the number of nucleotide sequence substitutions per site. The 20 reference phages included for comparison. The analysis included 73 nucleotide sequences.

**Comparative genomics (Figure S7-14)**

General description

Phage genome sequences were compared for each cluster (cluster A-F) using the progressiveMauve software. Boxes with same colours represent local collinear blocks (LCB), indicating homologous DNA regions shared by two or more genomes without sequence rearrangements. LCBs indicated below the horizontal black line represent reverse compliments of the reference LCB (reference genome is marked with a blue square). The height of the similarity profile within the LCBs corresponds to the average level of conservation in that region of the genome sequence. White boxes below the horizontal black line represents annotated genes in the reference sequences included. The terminase large subunit encoding genes is indicated with a black square in each genome sequence.


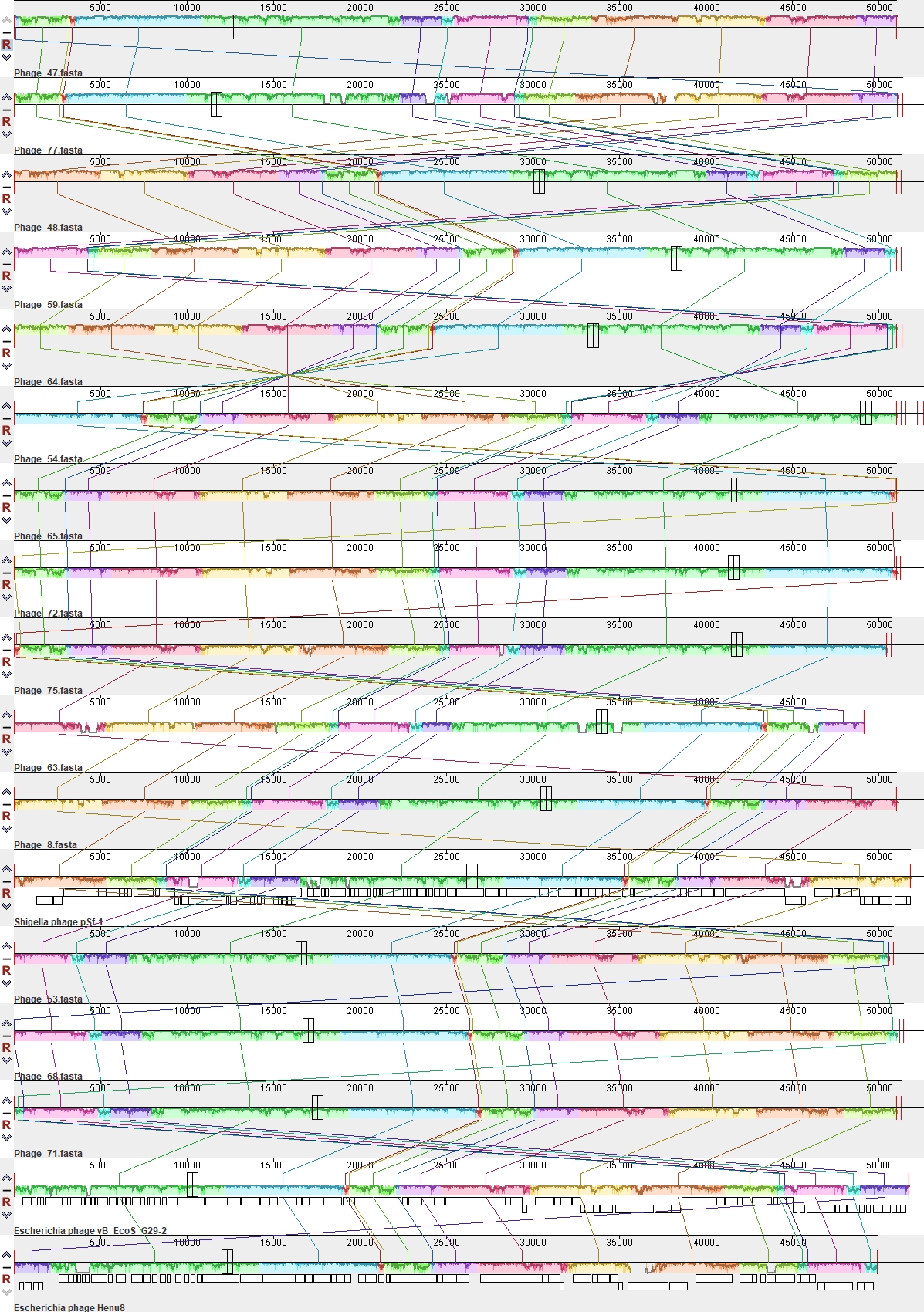


**Supplementary Figure S7** | **Comparative genomics of subcluster A1 phages.** Genome sequences of 17 *Siphoviridae* subcluster A1 phages were compared. 16 LCBs were identified.


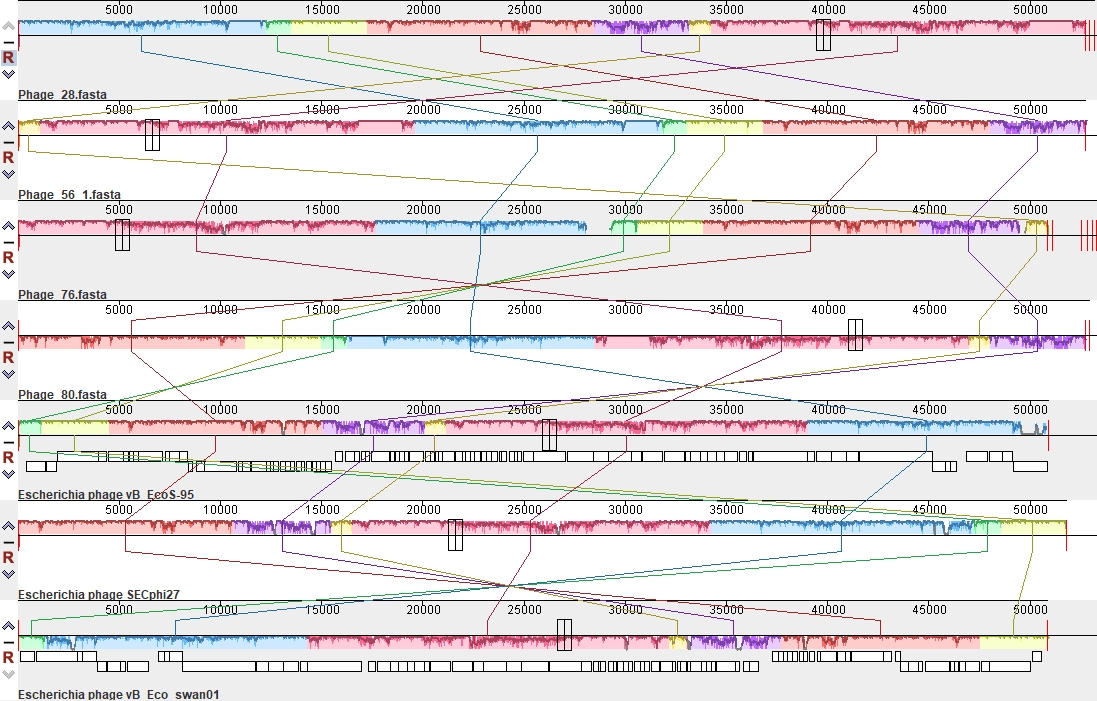


**Supplementary Figure S8** | **Comparative genomics of subcluster A2 phages**. Genome sequences of seven *Siphoviridae* subcluster A2 phages were compared. Seven LCBs were identified.


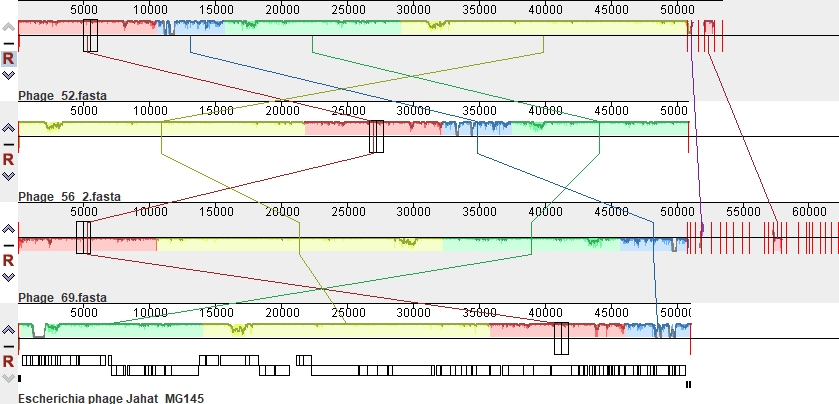
 **Supplementary Figure S9** | **Comparative genomics of subcluster A3 phages.** Genome sequences of four *Siphoviridae* subcluster A3 phages were compared. Four LBCs were identified.

**
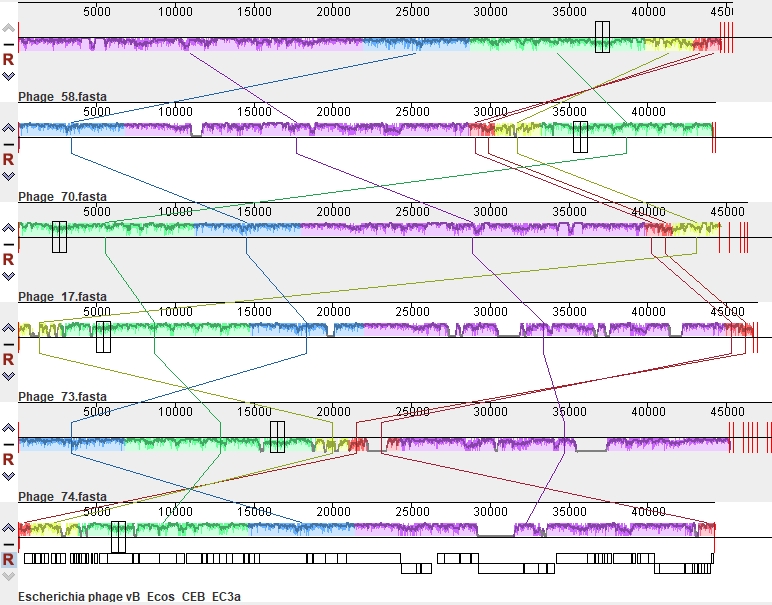
**

**Supplementary Figure S10** | **Comparative genomics of cluster B phages.** Genome sequences of six *Siphoviridae* cluster B phages were compared. Six LCBs were identified.

**
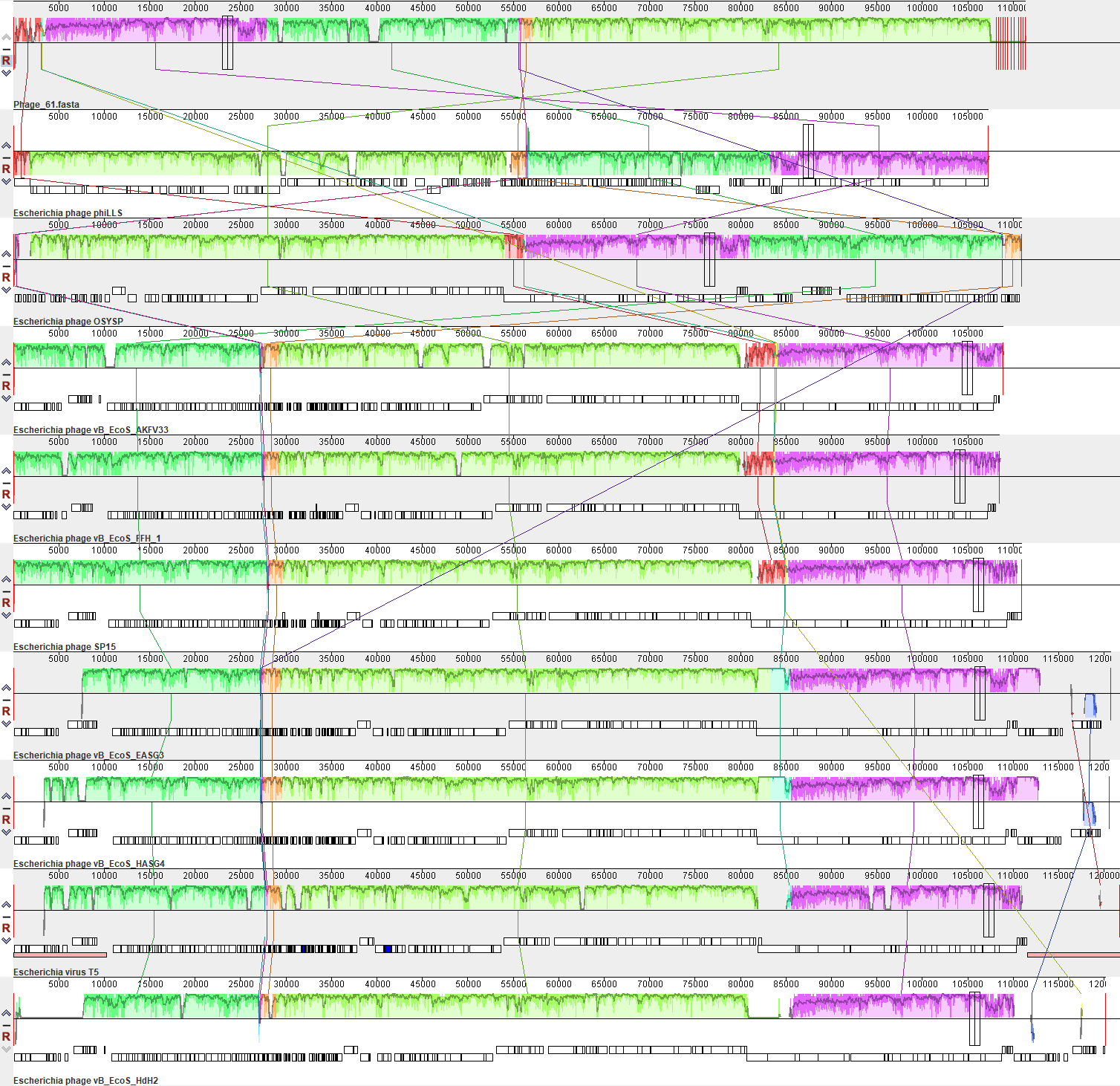
** **Supplementary Figure S11** | **Comparative genomics of cluster C phages. 10** Genome sequences of 10 *Siphoviridae* phages were compared. 6-10 LCBs were identified in each genome. Repeat-rich regions are indicated with salmon-coloured bars next to the white annotation boxes.


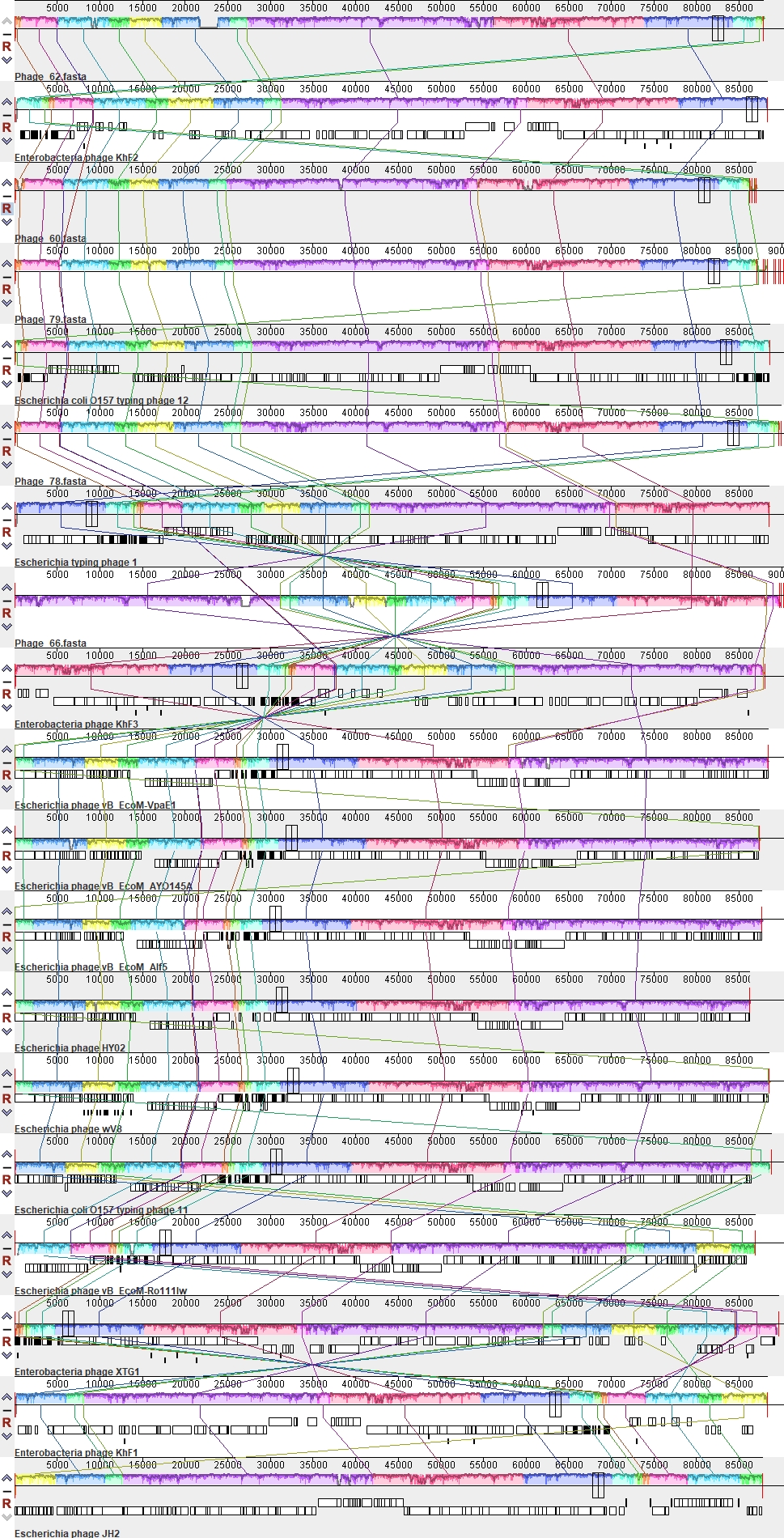


**Supplementary Figure S12** | **Comparative genomics of cluster D phages.** Genome sequences of 19 *Myoviridae* cluster D phages were compared. 14-17 LCBs were identified for each phage genome.


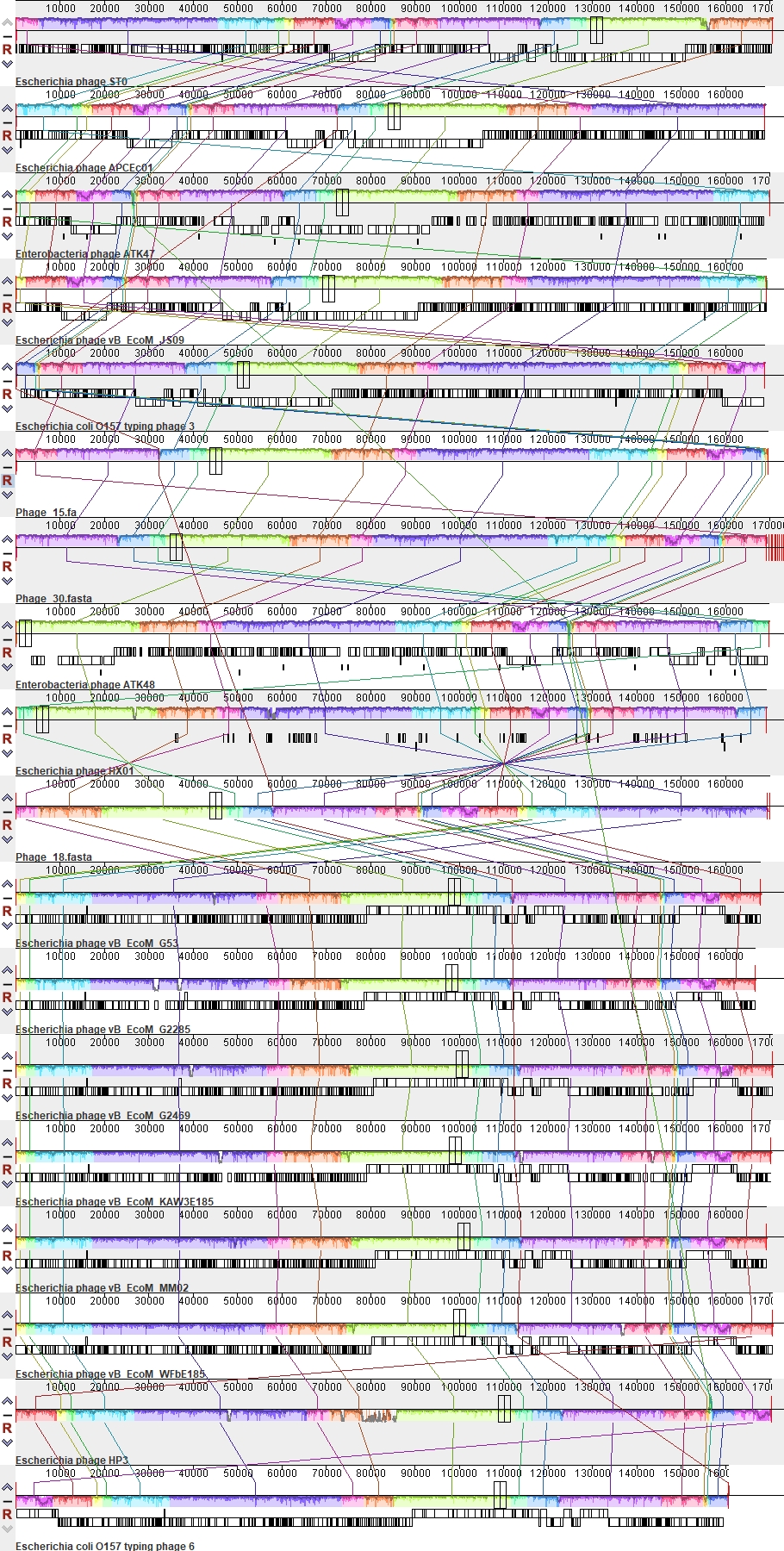


**Supplementary Figure S13** | **Comparative genomics of cluster E phages.** Genome sequences of 18 *Myoviridae* cluster E phages were compared. 16-17 LCBs were identified for each phage genome.

**
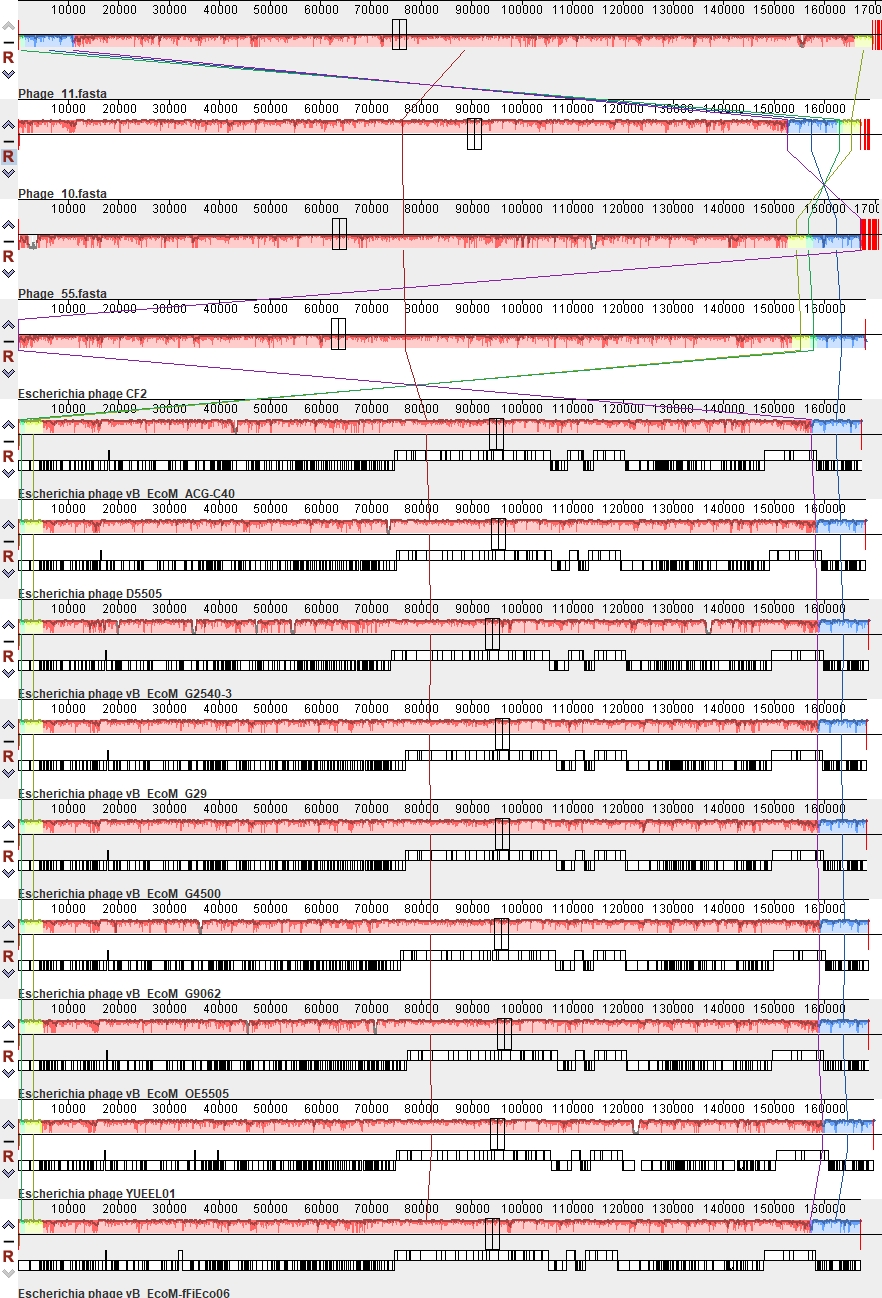
**

**Supplementary Figure S14** | **Comparative genomics of F subcluster phages.** Genome sequences of 13 *Myoviridae* cluster F phages were compared. Five LCBs were identified for each phage genome.
